# Physical Activity Throughout Adolescence and Peak Hip Strength in Young Adults

**DOI:** 10.1001/jamanetworkopen.2020.13463

**Published:** 2020-08-17

**Authors:** Ahmed Elhakeem, Jon Heron, Jon H. Tobias, Deborah A. Lawlor

**Affiliations:** 1 MRC Integrative Epidemiology Unit, University of Bristol, Bristol, United Kingdom; 2 Population Health Sciences, Bristol Medical School, University of Bristol, Bristol, United Kingdom; 3 Musculoskeletal Research Unit, Translational Health Sciences, Bristol Medical School, University of Bristol, Bristol, United Kingdom

## Abstract

**Importance:**

Peak bone strength, which occurs in early adulthood, is an important marker of the future risk of osteoporosis. It is therefore important to identify modifiable early life factors that are associated with the attainment of peak hip strength.

**Objective:**

To investigate the association of time spent in moderate to vigorous–intensity and light-intensity physical activity throughout adolescence with peak hip strength in adulthood.

**Design, Setting, and Participants:**

The Avon Longitudinal Study of Parents and Children is a prospective birth cohort study that initially recruited all pregnant women residing within the catchment area of 3 health authorities in southwest England who had an expected delivery date between April 1, 1991, and December 31, 1992. In total, 15 454 eligible pregnant women were enrolled, and 15 589 infants were delivered. Of those, 14 901 infants were alive at age 1 year. The present analysis examined 2569 healthy offspring who had valid physical activity measurements obtained during a clinical assessment for at least 1 age (12, 14, 16, and/or 25 years), with up to 4 repeated accelerometer assessments performed (1 per age-associated clinical visit). Data were analyzed from June 2019 to June 2020.

**Exposures:**

Trajectories of accelerometer-assessed time spent in moderate to vigorous–intensity and light-intensity physical activity at ages 12, 14, 16, and 25 years (measured in minutes per day) were identified using latent trajectory modeling. Moderate to vigorous–intensity and light-intensity physical activity were determined using established thresholds of acceleration counts per minute.

**Main Outcomes and Measures:**

Femur neck bone mineral density (BMD; measured in g/cm^2^) at age 25 years assessed by dual-energy radiography absorptiometry scans of the hip.

**Results:**

A total of 2569 participants (1588 female participants [62%]) were included in the analysis. Male participants spent more time in moderate to vigorous–intensity activity at each age and had greater adult femur neck BMD than female participants. For each sex, 3 moderate to vigorous–intensity trajectory subgroups and 3 light-intensity trajectory subgroups were identified. With regard to the moderate to vigorous–intensity trajectories, most male participants (85%) were in the low adolescent subgroup, with only 6% and 9% in the high early-adolescent and high mid-adolescent subgroups, respectively. Moderate to vigorous–intensity trajectories in female participants were divided into low adolescent-low adult (73%), low adolescent-high adult (8%), and high adolescent (19%) subgroups. Light-intensity physical activity trajectories were classified into low nonlinear, moderate decreasing, and high decreasing subgroups for both sexes. Femur neck BMD in male participants was greater in the high early-adolescent subgroup (0.38 g/cm^2^; 95% CI, 0.11-0.66 g/cm^2^) and the high mid-adolescent subgroup (0.33 g/cm^2^; 95% CI, 0.07-0.60 g/cm^2^) compared with the low adolescent (reference) subgroup. Femur neck BMD in female participants was greater in the high adolescent subgroup (0.28 g/cm^2^; 95% CI, 0.15-0.41 g/cm^2^) but not in the low adolescent-high adult subgroup (−0.12 g/cm^2^; 95% CI, −0.44 to 0.20 g/cm^2^) compared with the low adolescent-low adult (reference) subgroup. A sensitivity analysis using a negative-outcome control variable to explore unmeasured confounding supported these findings. The light-intensity trajectories were not associated with femur neck BMD; for example, differences in femur neck BMD between the high decreasing and low nonlinear subgroups were 0.16 g/cm^2^ (95% CI, −0.08 to 0.40 g/cm^2^) in male participants and 0.20 g/cm^2^ (95% CI, −0.05 to 0.44 g/cm^2^) in female participants.

**Conclusions and Relevance:**

Supporting high-intensity physical activity throughout early life may help to maximize peak hip strength and prevent osteoporosis in later life. Replication of our findings in independent studies will be important.

## Key Points

**Question:**

Is the amount of time spent in moderate to vigorous–intensity and light-intensity physical activity throughout adolescence associated with a clinical marker of hip strength in young adult men and women?

**Findings:**

In this cohort study of 2569 young people who received repeated accelerometer assessments beginning at age 12 years, more time spent in moderate to vigorous–intensity physical activity in adolescence was associated with greater hip bone mineral density at age 25 years, whereas more time spent in light-intensity physical activity was not associated with bone mineral density at age 25 years.

**Meaning:**

The findings indicate that higher-intensity physical activity in early life may be important for maximizing peak adult hip strength and protecting against osteoporosis in later life.

## Teaser

This cohort study of healthy offspring from the Avon Longitudinal Study of Parents and Children examines the association of time spent in moderate to vigorous–intensity and light-intensity physical activity throughout adolescence with peak hip strength in adulthood.

## Introduction

Peak bone strength occurs in early adulthood^1,2,3,4,5^ and is considered an important marker of bone strength, osteoporosis risk, and fracture risk in later life.^4,5,6,7,8^ Hip fractures compose a large proportion of the disease burden of osteoporosis^9,10,11,12^; thus, it is important to identify modifiable early life factors that have consequences for the attainment of peak hip strength. Data suggest that higher-intensity physical activity is beneficial for bone strength.^13,14,15,16,17,18,19,20,21,22,23,24,25,26^ Of the studies conducted among younger people, most were of young adolescents and examined activity at a single point or used self-reported data.^21,22,23,24,25,26^ Studies examining physical activity at a single point do not address the role that different patterns of change in or maintenance of physical activity has in bone strength and may be biased by regression to the mean.^27,28^ Self-reports are susceptible to errors and not well suited to capturing light-intensity activity.^29,30^ In addition to the role of higher-intensity activities in bone strength, studies suggest that activities producing higher gravitational force may be needed to strengthen bones.^31,32,33^ However, to our knowledge, the association between accelerometer-assessed gravitational force during physical activity and peak hip strength has not been examined.

The aim of this study was to investigate the association between accelerometer-measured moderate to vigorous–intensity and light-intensity physical activity trajectories beginning at age 12 years and hip strength at age 25 years. We also explored the association of gravitational force during physical activity measured by custom-built accelerometers at age 18 years with hip strength at age 25 years.

## Methods

### Study Population

**Figure 1.**
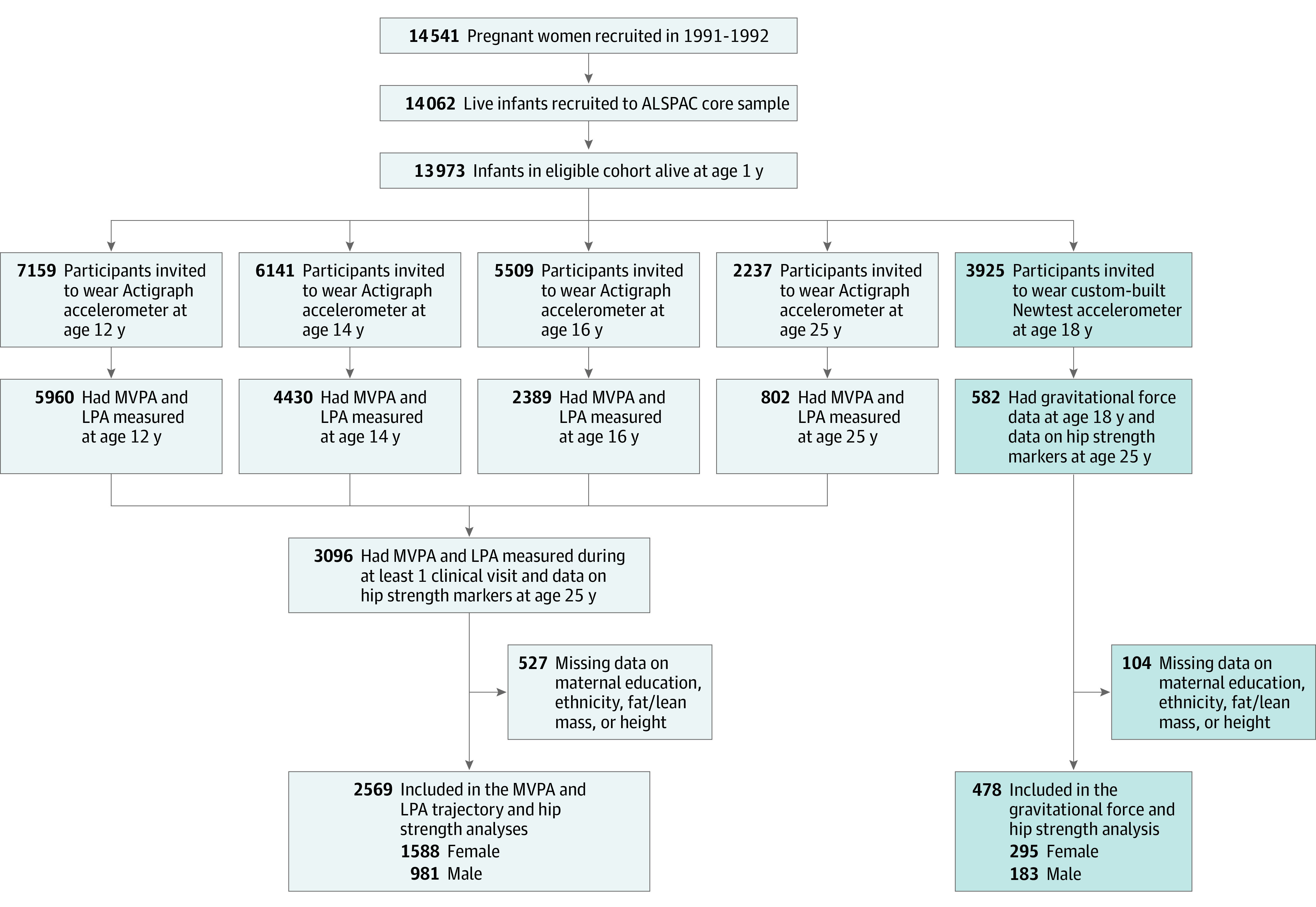
Study Flowchart ALSPAC indicates Avon Longitudinal Study of Parents and Children; LPA, light-intensity physical activity; and MVPA, moderate to vigorous–intensity physical activity.

The Avon Longitudinal Study of Parents and Children (ALSPAC) is a prospective birth cohort study that initially recruited all pregnant women residing within the catchment area of 3 health authorities in southwest England who had an expected delivery date between April 1, 1991, and December 31, 1992.^34,35,36^ In total, 15 454 eligible pregnant women (75% response rate) were enrolled in ALSPAC, and 15 589 infants were delivered. Of those, 14 901 infants were alive at age 1 year. Detailed information has been collected from index offspring and parents using questionnaires, data from linked health and social records, and clinical assessments up to the last completed contact in 2019. The present analysis examined 2569 healthy index offspring who had valid physical activity measurements obtained during a clinical assessment for at least 1 age (12, 14, 16, and/or 25 years), with up to 4 repeated accelerometer assessments performed (1 per age-associated clinical visit). Participants with missing covariate data (527 of 3096 individuals [18%] who were potentially eligible) were excluded. Details of all available data can be found at the ALSPAC study website,^37^ which includes a searchable data dictionary and variable search tool. A flowchart of participant selection for the present analysis is shown in Figure 1.

Ethical approval was obtained from the ALSPAC Ethics and Law Committee and the local research ethics committees. Full details of ethics committee approvals can be found on the study website.^37^ Written informed consent was obtained from all participants. This study followed the Strengthening the Reporting of Observational Studies in Epidemiology (STROBE) reporting guideline for cohort studies.

### Physical Activity Intensity

All offspring who attended clinical assessments at ages 12, 14, 16, and 25 years were asked to wear an AM7164 accelerometer (Actigraph) for 7 days during waking hours and to remove the accelerometer only when showering, bathing, and performing water sports.^38,39,40,41,42^ These devices capture movement in terms of acceleration as a combined function of frequency and intensity. Data were processed using Kinesoft software, version 3.3.75 (Kinesoft), according to a predefined protocol described elsewhere.^41,43^

The analysis was restricted to participants with 3 or more days of valid data (≥500 minutes per day, after excluding intervals of ≥60 minutes of 0 counts). Activity counts per minute thresholds validated in young people^44^ and adults^45^ were used to calculate the amount of time spent in moderate to vigorous–intensity and light-intensity physical activity throughout adolescence (ie, at ages 12, 14, and 16 years; for moderate to vigorous–intensity activity, >2296 counts per minute; for light-intensity activity, 100-2296 counts per minute) and in adulthood (ie, at age 25 years; for moderate to vigorous–intensity activity, >2020 counts per minute; for light-intensity activity, 100-2020 counts per minute).

### Physical Activity Gravitational Force

At the clinical assessment for age 18 years, a subgroup of participants (depending on device availability) was fitted with a custom-built accelerometer (Newtest; Newtest Oy), which was used to explore the association between gravitational force during physical activity and bone health. All participants in this subgroup had previously worn an Actigraph accelerometer during at least 1 clinical assessment for at least 1 age, (ie, age 12, 14, and/or 16 years).

The Newtest device recorded gravitational force from vertical accelerations within separate bands across the range of 0.3*g* to 9.9*g* above the conventional value of gravitational acceleration (ie, 1.0*g*, or approximately 9.8 m/s^2^). Participants were asked to wear the device for 7 consecutive days during waking hours, recharge it overnight, and remove it only for contact sports or for situations in which it might get wet. A valid recording was defined as 8 or more hours of recording per day for 2 or more days.^31^ For this study, gravitational force was expressed as counts across 4 bands (0.5*g* to ≤1.1*g*, >1.1*g* to ≤3.1*g*, >3.1*g* to ≤5.1*g*, and >5.1*g*). These bands represent gravitational force from movements, such as normal walking (0.5*g *to ≤1.1*g*) and jumping (>5.1*g*), as determined by previous studies.^46,47,48,49^

### Adult Hip Strength Assessment

All participants were invited to receive dual-energy radiography absorptiometry scans of the hip as part of the clinical assessment at age 25 years. Scans were performed between June 2015 and October 2017 using the same scanner (GE Lunar Prodigy; GE Healthcare) for all participants. All scans were performed on the left hip and were repeated if correct alignment was not achieved. Scans were analyzed using the manufacturer’s standard scanning software and positioning protocols. A total of 50 scans with artifacts, positioning errors, incorrect neck or shaft angles, missing hip parts, or high room temperature (>27 °C) were excluded.

Total hip and femoral neck bone mineral density (BMD; measured in g/cm^2^) were generated from the scans.^50^ Bone mineral density is the criterion standard for diagnosing osteoporosis in clinical practice,^51^ but it only provides information on the quantity of bone tissue.^52^ Because bone strength is a function of both the quantity and quality of bone tissue,^52^ and bone geometry is associated with bone quality and strength,^52,53,54,55,56,57^ we used the manufacturer's automated hip analysis software to derive 4 hip geometric parameters (minimum femur neck width [measured in mm], cross-sectional area [measured in mm^2^], section modulus [measured in mm^3^], and cross-sectional moment of inertia [measured in mm^4^]) as additional outcomes.

### Confounding Variables

Childhood socioeconomic position, ethnicity, height, adiposity, and muscle mass were defined a priori as potential confounding variables based on the assumption that they were associated with both adolescent physical activity and adult hip strength.^58^ These factors were all assessed before the first Actigraph accelerometer assessment. Self-reported maternal socioeconomic position (highest educational level [≥college degree vs <college degree]) and maternal ethnicity (White with European ancestry vs other ethnicity) were obtained at recruitment (ie, during pregnancy).

Childhood height, adiposity, and muscle mass were measured during the clinical assessment at age 10 years by accredited field workers. Height was measured without shoes, with the head in the correct position, using a stadiometer (Harpenden; Holtain). Fat (adiposity) and lean (muscle) mass were obtained from whole-body dual-energy radiography absorptiometry scans. Height-adjusted indices were calculated by dividing mass in kilograms by height in meters^1,2,59^

### Statistical Analysis

We performed latent trajectory modeling^40,60,61,62,63^ using Mplus software, version 8 (Muthen & Muthen), to identify sex-specific trajectories of moderate to vigorous–intensity and light-intensity physical activity from age 12 to 25 years. These models aim to classify individuals into distinct subgroups that share similar trajectories over time, such that individuals within a group are more similar than individuals between groups. Modeling was conducted according to published guidelines^62,63^ and is detailed in eMethods, eTable 1 to eTable 6, and eFigure 1 to eFigure 12 in the Supplement.

A linear regression analysis was used to estimate the association of derived moderate to vigorous–intensity and light-intensity trajectory subgroups with hip strength markers at age 25 years. Linear regression models were also used to explore the associations between vertical acceleration counts within each gravitational force band at age 18 years and hip strength markers at age 25 years. Counts were log-transformed to minimize skew; estimates were reported as differences in outcomes per doubling in the number of force measurements. Both unadjusted and adjusted (for all confounding variables) models were fitted for each outcome. Data were analyzed from June 2019 to June 2020.

### Sensitivity Analysis

We assessed whether the associations of physical activity with hip strength were robust to uncontrolled confounding by performing a negative-outcome control analysis.^64,65^ Detailed descriptions of the rationale for performing negative-outcome control analyses and our choice of negative-outcome control variable are available in eMethods, eTable 7, and eFigure 13 in the Supplement.

In brief, an ideal negative-outcome control would share the same confounding variables (measured or unmeasured) as adult hip strength but would not plausibly be associated with adolescent physical activity.^64,65^ For this study, adult leg length (calculated by subtracting seated height from standing height at age 25 years) was used as a negative-outcome control. Because leg length is sensitive to early-life environments,^66,67,68^ it likely shares similar early life factors with hip strength; however, an association between physical activity (intensity or gravitational force) across adolescence and adult leg length seemed unlikely. Therefore, any association with adult leg length would likely be owing to confounding and suggests the same may be true for the hip strength analyses.

## Results

Among 2569 participants included in the analysis, 1588 individuals (62%) were female and 981 individuals (38%) were male. Among both sexes, the mean (SD) ages at the adolescent clinic visits were 11.7 (0.2) years at the assessment for age 12 years, 13.8 (0.2) years at the assessment for age 14 years, and 15.4 (0.3) years at the assessment for age 16 years. All of the participants had valid physical activity measurements that were obtained during a clinical assessment for at least 1 age (6140 moderate to vigorous–intensity and light-intensity activity measurements in total, with a median of 2 measurements [interquartile range, 1-3 measurements] per individual) and complete data on hip outcomes and confounding variables (Table).

**Table.** Characteristics of Participants Included in the Trajectory Analyses

Characteristic	Mean (SD)	
	Male participants (n = 981)	Female participants (n = 1588)
Age at Actigraph accelerometer assessment, y		
12	11.7 (0.2)	11.7 (0.2)
14	13.8 (0.2)	13.8 (0.2)
16	15.4 (0.3)	15.4 (0.3)
25	24.5 (0.8)	24.4 (0.8)
Moderate to vigorous–intensity physical activity min/d at each age (counts/min)		
12 y (>2295)	65.1 (28.5)	45.4 (19.8)
14 y (>2295)	58.9 (28.1)	43.4 (22.3)
16 y (>2295)	54.9 (30.4)	38.6 (21.4)
25 y (>2020)	54.2 (33.0)	46.4 (27.1)
Light-intensity physical activity min/d at each age (counts/min)		
12 y (100-2295)	366.3 (61.1)	363.0 (59.4)
14 y (100-2295)	327.7 (63.3)	308.1 (60.1)
16 y (100-2295)	285.9 (67.9)	269.1 (62.5)
25 y (100-2020)	148.6 (60.6)	148.5 (53.5)
Hip strength markers at age 25 y		
Bone mineral density, g/cm^2^		
Total hip	1.13 (0.2)	1.05 (0.1)
Femur neck	1.11 (0.2)	1.04 (0.1)
Femur minimum neck width, mm	33.9 (2.7)	28.6 (2.1)
Cross-sectional area, mm^2^	186.9 (31.1)	150.0 (21.6)
Section modulus, mm^3^	920.4 (199.4)	629.1 (117.3)
Cross-sectional moment of inertia, mm^4^	16 572 (4366)	9412 (2285)
Early-life anthropometry and body composition at age 10 y		
Height, cm	140.1 (6.0)	138.9 (6.3)
Fat mass index, kg/m^1.2^	4.8 (3.0)	6.2 (3.0)
Lean mass index, kg/m^1.2^	17.0 (1.3)	15.7 (1.4)
Ethnicity, No. (%)		
White European	963 (98)	1554 (98)
Other	18 (2)	34 (2)
Maternal educational level, No. (%)		
≥College degree	233 (24)	330 (21)
<College degree	748 (76)	1258 (79)

Male participants compared with female participants spent more time in moderate to vigorous activity at each age (eg, at age 12 years, the mean [SD] level of moderate to vigorous physical activity was 65.1 [28.5] minutes per day in male participants vs 45.4 [19.8] minutes per day in female participants) and had greater adult hip BMD (eg, mean [SD] total hip BMD was 1.13 [0.2] g/cm^2^ in male participants vs 1.05 [0.1] g/cm^2^ in female participants) and geometric parameters (eg, mean [SD] femur minimum neck width was 33.9 [2.7] mm in male participants vs 28.6 [2.1] mm in female participants) (Table). Overall, between age 12 and 25 years, the levels of light-intensity physical activity decreased with age in both male participants (mean [SD], 366.3 [61.1] minutes per day at age 12 years vs 148.6 [60.6] minutes per day at age 25 years) and female participants (mean [SD], 363.0 [59.4] minutes per day at age 12 years vs 148.5 [53.5] minutes per day at age 25 years). The level of moderate to vigorous–intensity activity decreased in male participants throughout adolescence (mean [SD], 65.1 [28.5] minutes per day at age 12 years vs 54.9 [30.4] minutes per day at age 16 years), remained stable in female participants through early adolescence (mean [SD], 45.4 [19.8] minutes per day at age 12 years vs 43.4 [22.3] minutes per day at age 14 years), and increased in female participants at age 25 years (mean [SD], 38.6 [21.4] minutes per day at age 16 years vs 46.4 [27.1] minutes per day at age 25 years) (Table; eFigure 14 in the Supplement). Additional early-life characteristics of study participants are shown in the Table.

### Physical Activity Intensity Trajectories

**Figure 2.**
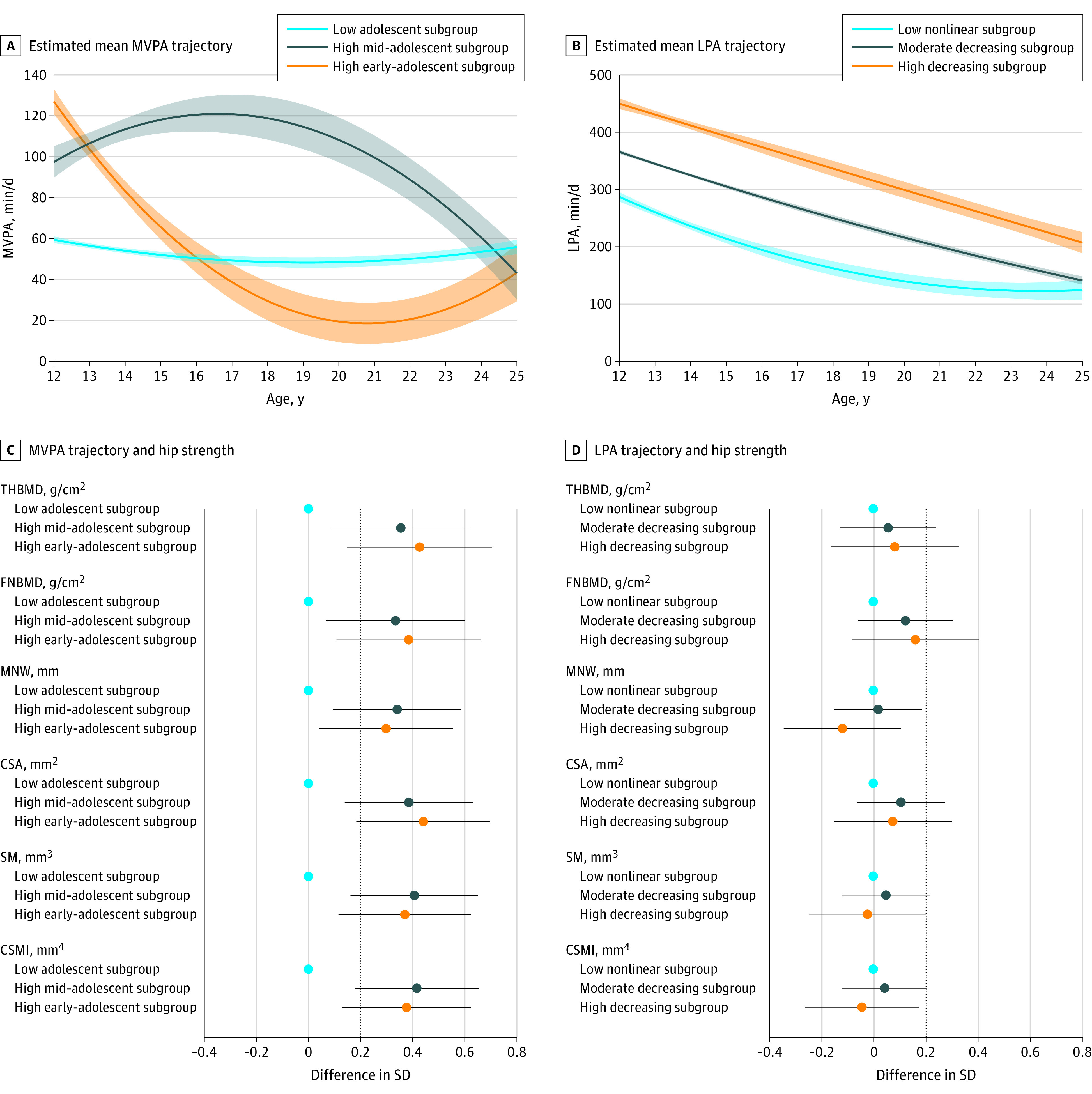
Association of Moderate to Vigorous–Intensity and Light-Intensity Physical Activity Trajectories With Hip Strength Markers in Male Participants Estimates are adjusted for ethnicity, maternal education, childhood height, fat and lean mass indices, and age at hip scan. CSA indicates cross-sectional area; CSMI, cross-sectional moment of inertia; FNBMD, femur neck bone mineral density; LPA, light-intensity physical activity; MNW, minimum neck width; MVPA, moderate to vigorous–intensity physical activity; SM, section modulus; and THBMD, total hip bone mineral density. A, Estimated mean time spent in moderate to vigorous–intensity activity. Shaded areas surrounding mean trajectories represent 95% CIs. B, Estimated mean time spent in light-intensity physical activity. Shaded areas surrounding mean trajectories represent 95% CIs. C, Difference in hip strength markers at age 25 years for moderate to vigorous–intensity activity trajectory subgroup. The low adolescent subgroup was the reference group. Horizontal bars represent 95% CIs. D, Difference in hip strength markers at age 25 years for light-intensity activity trajectory subgroup. The low nonlinear subgroup was the reference group. Horizontal bars represent 95% CIs.

We identified 3 activity trajectory subgroups in male and female participants for both moderate to vigorous–intensity and light-intensity physical activity. Among male participants, the 3 moderate to vigorous–intensity trajectory subgroups had notably different mean amounts of time spent in this activity at age 12 years (Figure 2A). The mean amount of time spent in moderate to vigorous activity decreased as age increased to 25 years in the group with the highest level of time spent in this activity at age 12 years (6%). The mean amount of time spent in moderate to vigorous activity increased throughout adolescence and decreased at age 25 years in the group with the second-highest level of time spent in this activity at age 12 years (9%). The group of male participants with the least amount of time spent in moderate to vigorous activity at age 12 years (85%) had a pattern of decreasing levels of time spent in this activity throughout adolescence and a small increase in the level of time spent in this activity at age 25 years. We named these 3 subgroups high early-adolescent moderate to vigorous–intensity physical activity, high mid-adolescent moderate to vigorous–intensity physical activity, and low adolescent moderate to vigorous–intensity physical activity.

**Figure 3.**
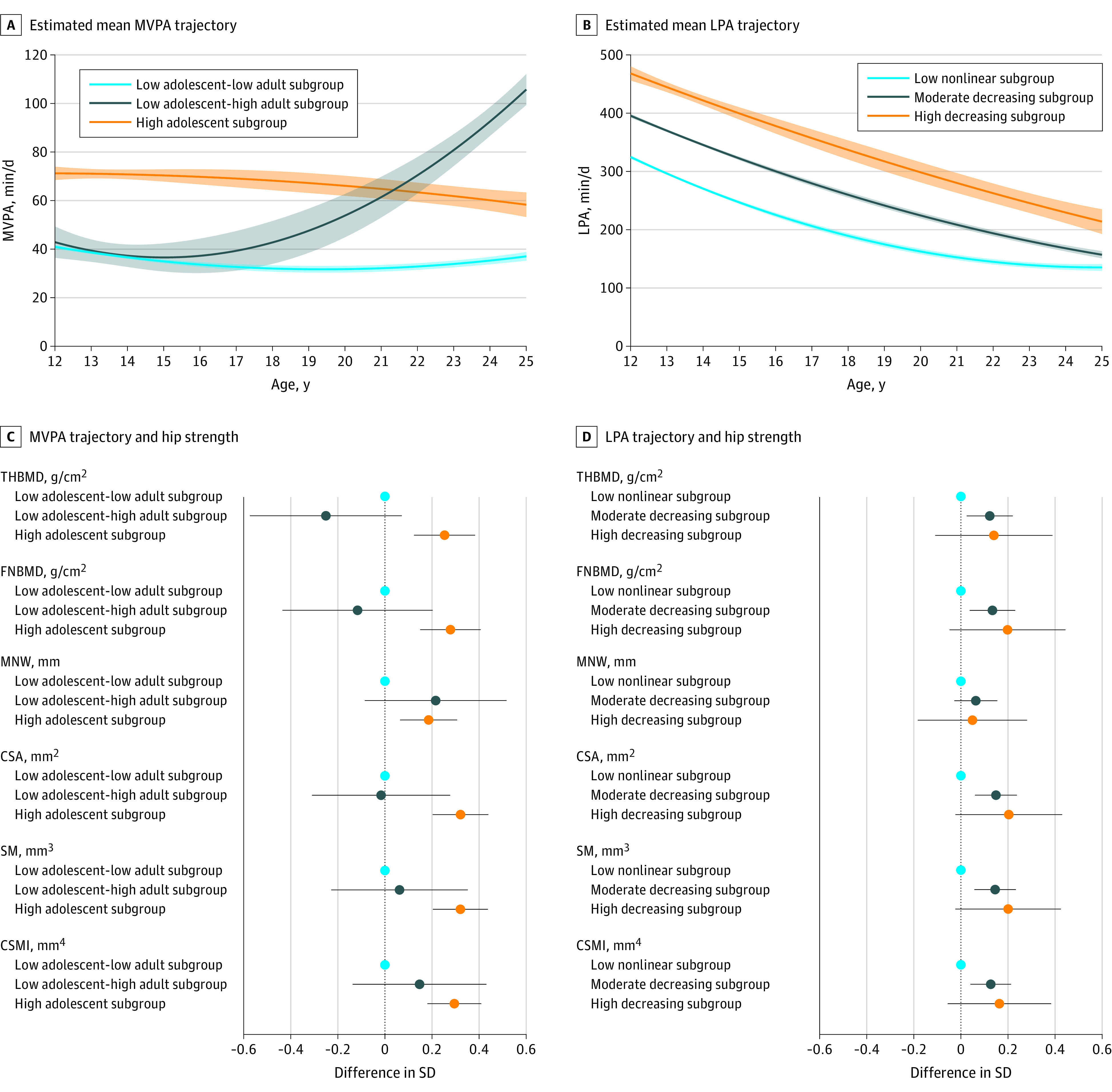
Association of Moderate to Vigorous–Intensity and Light-Intensity Physical Activity Trajectories With Hip Strength Markers in Female Participants Estimates are adjusted for ethnicity, maternal education, childhood height, fat and lean mass indices, and age at hip scan. CSA indicates cross-sectional area; CSMI, cross-sectional moment of inertia; FNBMD, femur neck bone mineral density; LPA, light-intensity physical activity; MNW, minimum neck width; MVPA, moderate to vigorous–intensity physical activity; SM, section modulus; and THBMD, total hip bone mineral density. A, Estimated mean time spent in moderate to vigorous–intensity activity. Shaded areas surrounding mean trajectories represent 95% CIs. B, Estimated mean time spent in light-intensity physical activity. Shaded areas surrounding mean trajectories represent 95% CIs. C, Difference in hip strength markers at age 25 years for moderate to vigorous–intensity activity trajectory subgroup. The low adolescent-low adult subgroup was the reference group. Horizontal bars represent 95% CIs. D, Difference in hip strength markers at age 25 years for light-intensity activity trajectory subgroup. The low nonlinear subgroup was the reference group. Horizontal bars represent 95% CIs.

Among female participants, 1 trajectory subgroup had a notably higher mean amount of time spent in moderate to vigorous activity at age 12 years compared with the 2 other subgroups (Figure 3A). This subgroup (19%) maintained higher levels of time spent in moderate to vigorous–intensity activity throughout adolescence and had slightly decreased level of time spent in this activity at age 25 years. The other 2 trajectory subgroups had similarly low levels of time spent in moderate to vigorous activity at age 12 years and throughout adolescence. The smallest of these groups (8%) had the highest overall levels of time spent in moderate to vigorous activity at age 25 years, whereas the last subgroup (73%) had the lowest overall levels of time spent in moderate to vigorous activity at age 25 years. We named these subgroups high adolescent moderate to vigorous–intensity physical activity, low adolescent-high adult moderate to vigorous–intensity physical activity, and low adolescent-low adult moderate to vigorous–intensity physical activity.

Among both male and female participants, the 3 similar light-intensity trajectory subgroups had notably different mean levels of time spent in light-intensity activity at age 12 years (Figure 2B and Figure 3B). The mean levels of time spent in light-intensity activity decreased monotonically with increasing age to 25 years in the 2 subgroups with the highest and second-highest levels of time spent in light-intensity activity at age 12 years such that, by age 25 years, the difference in time spent in light-intensity activity between these 2 groups was similar to that observed at age 12 years. Those spending the least time in LPA at age 12 showed a pattern of decreasing time spent through adolescence, after which mean time spent in LPA increased. We named these 3 subgroups high decreasing light-intensity physical activity, moderate decreasing light-intensity physical activity, and low nonlinear light-intensity physical activity. Most male participants were in the moderate decreasing subgroup (67%), with a similar proportion of the remaining male participants in the high decreasing and low nonlinear trajectory subgroups. In comparison with male participants, most female participants were in either the low nonlinear subgroup (51%) or the moderate decreasing (43%) subgroup.

### Physical Activity Intensity Trajectories and Adult Hip Strength

Among the moderate to vigorous–intensity trajectory subgroups, the mean adult hip BMD and geometric parameters in male participants were all notably higher in the high early-adolescent (eg, femur neck BMD, 0.38 g/cm^2^ [95% CI, 0.11-0.66 g/cm^2^]; total hip BMD, 0.43 g/cm^2^ [95% CI, 0.15-0.71 g/cm^2^]) and high mid-adolescent (eg, femur neck BMD, 0.33 g/cm^2^ [95% CI, 0.07-0.60 g/cm^2^]; total hip BMD, 0.35 g/cm^2^ [95% CI, 0.09-0.62 g/cm^2^]) subgroups compared with the low adolescent (reference) subgroup (Figure 2C). Estimates were similar for both of these moderate to vigorous–intensity activity groups. In female participants, adult hip BMD and geometric parameters were higher in the high adolescent subgroup (eg, femur neck BMD, 0.28 g/cm^2^ [95% CI, 0.15 to 0.41 g/cm^2^]; total hip BMD, 0.25 g/cm^2^ [95% CI, 0.12-038 g/cm^2^]) but not in the low adolescent-high adult subgroup (eg, femur neck BMD, −0.12 g/cm^2^ [95% CI, −0.44 to 0.20 g/cm^2^]; total hip BMD, -0.25 g/cm^2^ [95% CI, −0.57 to 0.07 g/cm^2^]) compared with the low adolescent-low adult (reference) subgroup (Figure 3C). There was no difference in adult hip strength markers between the low adolescent-high adult and low adolescent-low adult subgroups (eFigure 15 in the Supplement).

An association between light-intensity activity trajectories and adult hip strength parameters was less consistently observed. In male participants, the mean adult hip BMD and geometric parameters in the high decreasing subgroup (eg, femur neck BMD, 0.16 g/cm^2^ [95% CI, −0.08 to 0.40 g/cm^2^]; total hip BMD, 0.08 g/cm^2^ [95% CI, −0.16 to 0.33 g/cm^2^]) and the moderate decreasing subgroup (eg, femur neck BMD, 0.12 g/cm^2^ [95% CI, −0.06 to 0.30 g/cm^2^]; total hip BMD, 0.06 g/cm^2^ [95% CI, −0.13 to 0.24 g/cm^2^]) were similar to those of the low nonlinear (reference) subgroup (Figure 2D). In female participants, the mean adult hip BMD and geometric parameters were higher in both the high decreasing subgroup (eg, femur neck BMD, 0.20 g/cm^2^ [95% CI, −0.05 to 0.44 g/cm^2^]; total hip BMD, 0.14 g/cm^2^ [95% CI, −0.11 to 0.39 g/cm^2^]) and the moderate decreasing subgroup (eg, femur neck BMD, 0.13 g/cm^2^ [95% CI, 0.04-0.23 g/cm^2^]; total hip BMD, 0.12 g/cm^2^ [95% CI, 0.02-0.22 g/cm^2^]) compared with the low nonlinear (reference) subgroup (Figure 3D). Results from unadjusted and adjusted models are presented in eTable 9 and eTable 10 in the Supplement. There was no difference between light-intensity trajectory subgroups for adult leg length (negative-outcome control variable) (eFigure 15 in the Supplement).

### Physical Activity Gravitational Force

**Figure 4.**
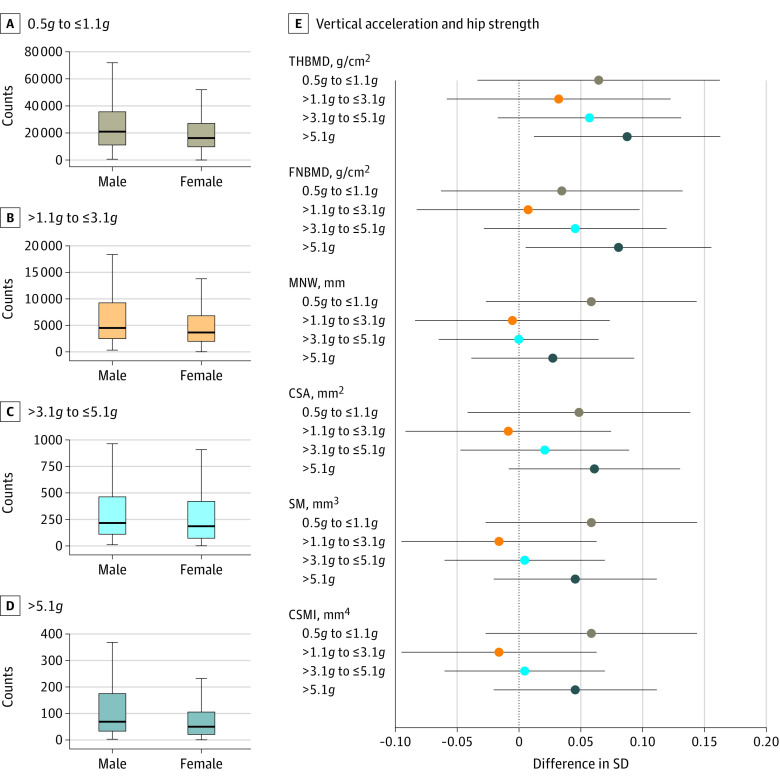
Association of Gravitational Force Measurements During Physical Activity With Hip Strength Markers Estimates are adjusted for sex, ethnicity, maternal education, childhood height, fat and lean mass indices, and age at hip scan. CSA indicates cross-sectional area of hip; CSMI, cross-sectional moment of inertia of hip; FNBMD, femur neck hip bone mineral density; MNW, minimum femur neck width; SM, section modulus of hip; and THBMD, total hip bone mineral density. A, Distribution of vertical acceleration counts in 0.5*g* to ≤1.1*g* band at age 18 years. B, Distribution of vertical acceleration counts in >1.1*g* to ≤3.1*g* band at age 18 years. C, Distribution of vertical acceleration counts in >3.1*g* to ≤5.1*g* band at age 18 years. D, Distribution of vertical acceleration counts in >5.1*g* band at age 18 years. For panels A through D, middle lines indicate the median, ends of boxes indicate the upper and lower quartiles, and whiskers indicate the range. E, Difference in hip strength markers at age 25 years. Difference per doubling in number of gravitational force measurements recorded in each band. Horizontal bars indicate 95% CIs.

A total of 478 participants (183 male participants and 295 female participants) with vertical gravitational force measurements recorded at age 18 years and complete data on adult hip outcomes and confounding variables were included in the analysis of this exposure (Figure 1; eTable 8 in the Supplement). Most gravitational force measurements were low in magnitude; only 58 of 23 923 registered measurements (0.2%) were greater than 5.1g (Figure 4A, Figure 4B, Figure 4C, and Figure 4D).

Despite their rarity, gravitational force measurements greater than 5.1*g* were positively associated with peak adult hip BMD and geometric parameters (Figure 4E). Positive associations with adult hip strength markers were observed for low gravitational force measurements (0.5*g* to ≤1.1*g*), whereas the association of medium gravitational force measurements (>1.1*g* to ≤3.1*g* and >3.1*g* to ≤5.1*g*) with hip strength markers was closer to the null. Numerical results from unadjusted and adjusted models are presented in eTable 11 in the Supplement. Force measurements greater than 5.1*g* were not associated with adult leg length (negative-outcome control variable) (eFigure 15 in the Supplement).

## Discussion

We used repeated accelerometer assessments of participants beginning at age 12 years to identify trajectories of the amount of time spent in moderate to vigorous–intensity and light-intensity physical activity throughout adolescence, and we investigated their associations with hip strength markers at age 25 years. A greater amount of time spent in moderate to vigorous–intensity activity during adolescence was associated with substantial and favorable differences in hip BMD and geometric parameters, whereas these associations were not consistently observed for the amount of time spent in light-intensity activity during adolescence. Exploratory analyses using custom-built accelerometers worn by participants at age 18 years indicated that, despite being rare, exposure to high-magnitude gravitational force was positively associated with hip strength. Our negative-outcome control sensitivity analysis suggests these findings are unlikely to be fully explained by uncontrolled confounding.

Our finding of an association between hip BMD and geometric parameters and moderate to vigorous–intensity activity, but not light-intensity activity, expands on previous accelerometer-based cross-sectional studies reporting that time spent in moderate to vigorous activity was positively associated with hip BMD and geometry.^14,24^ The findings also complement reported associations between consistent participation in organized sports from ages 5 to 17 years and greater leg BMD at age 20 years.^25^ Our results from female participants indicate that moderate to vigorous activity during adolescence is more important for adult hip strength than the participant’s current participation in moderate to vigorous activity, which is consistent with the hypothesis that adolescence is a sensitive period for bone development,^69^ particularly given data indicating that bone accrues rapidly during puberty.^1,50,70^ Furthermore, our findings from male participants indicate that both the early- and mid-adolescent moderate to vigorous–intensity subgroups were also associated with adult hip parameters, despite the early adolescent trajectory subgroup having a substantially greater decrease in moderate to vigorous activity. This finding suggests that moderate to vigorous–intensity physical activity may be more important in early adolescence than in later adolescence, which is consistent with data indicating that younger prepubertal skeletons are more responsive to mechanical loading from physical activity.^71^

The association found between high-magnitude gravitational force measurements in late adolescence and peak hip BMD and geometric parameters extends the previous cross-sectional results from ALSPAC^31^ and is consistent with self-reported data indicating that replacing low-impact activities with high-impact activities in childhood is associated with increased hip BMD.^23^ However, because our findings were derived from limited high-magnitude impact observations in a relatively small sample, the conclusions that can be drawn are limited. Nevertheless, when taken together, the results of our study suggest that moderate to vigorous–intensity physical activity (vs light-intensity activity) and higher gravitational force measurements (vs lower gravitational force measurements) throughout early life are associated with increases in bone mass during growth.^20,21,22^ These increases may be owing to direct osteogenic mechanisms and the indirect implications of high-intensity and high-impact activities for bone through the associated increases in lean mass.^71,72,73,74^

### Limitations

Participants with missing covariate data (18% of those potentially eligible) were excluded, which might have introduced bias if the excluded participants had systematically different hip measurements. Participants missing all accelerometry assessments were also excluded, and these participants had socioeconomic differences from the analytical sample, which might limit the generalizability of our findings. Participants with 1 or more measurement of moderate to vigorous–intensity or light-intensity activity were included in the latent trajectory models under the missing-at-random assumption, which cannot be fully tested. However, the probability of missing accelerometer data was associated with model confounders, which suggests that these data may be consistent with the missing-at-random assumption. Latent trajectory modeling is an important strength of the present study; however, these models can be data-specific, meaning that data from identified subgroups may not replicate in other cohorts, which limits their generalizability. Our sample mostly comprised White individuals of European ancestry, which might limit the study’s generalizability to individuals of other ethnicities. While these associations cannot be interpreted as causal, our negative-outcome control sensitivity analysis provides some indication that the findings are not fully explained by uncontrolled confounding.

## Conclusions

This prospective cohort study indicated that a greater amount of time spent in moderate to vigorous–intensity physical activity from age 12 years and a greater exposure to higher-magnitude gravitational force at age 18 years were associated with greater hip strength at age 25 years. Our findings suggest that higher-intensity physical activity, along with potential bursts of higher-impact activity, throughout adolescence may be important for maximizing peak hip strength during early adulthood. If replicated in independent studies, these findings suggest that children’s involvement in moderate to vigorous–intensity physical activity^75^ may be beneficial for lasting bone health.

## Notes

Supplement. **eMethods.** Latent Trajectory Modeling and Sensitivity Analysis for Uncontrolled Confounding **eTable 1.** Characteristics of the Initial Latent Trajectory Models With Varying Number of Classes **eTable 2.** Characteristics of the 3-Class Latent Trajectory Models With Varying Internal Model Structure **eTable 3.** Results of the Final 3-Class MVPA and LPA Latent Trajectory Models: Male Participants **eTable 4.** Results of the Final 3-Class MVPA and LPA Latent Trajectory Models: Female Participants **eTable 5.** Comparison Between Participants Included in Main Analysis With MVPA/LPA Accelerometer Assessments at All 4 Ages and Participants Included in Main Analysis With At Least 1 Missing MVPA/LPA Accelerometer Assessment **eTable 6.** Comparison Between Participants Included in Main Analysis and Participants Excluded From Main Analysis Due to Missing All 4 MVPA/LPA Accelerometer Assessments **eTable 7.** Association of Model Covariates With Adult Hip Strength Markers and Leg Length **eTable 8.** Characteristics of ALSPAC Participants Included in the Vertical Impact Analysis **eTable 9.** Associations of MVPA and LPA Trajectory With Hip Strength Markers: Male Participants **eTable 10.** Associations of MVPA and LPA Trajectory With Hip Strength Markers: Female Participants **eTable 11.** Association of Physical Activity Gravitational Impacts With Hip Strength Markers **eFigure 1.** Latent Trajectory Model Conceptualizations **eFigure 2.** MVPA Trajectories From Initial Latent Trajectory Models With Varying Number of Classes: Male Participants **eFigure 3.** LPA Trajectories From Initial Latent Trajectory Models With Varying Number of Classes: Male Participants **eFigure 4.** MVPA Trajectories From Initial Latent Trajectory Models With Varying Number of Classes: Female Participants **eFigure 5.** LPA Trajectories From Initial Latent Trajectory Models With Varying Number of Classes: Female Participants **eFigure 6.** MVPA Trajectories From 3-Class MVPA Latent Trajectory Models With Varying Internal Model Structure: Male Participants **eFigure 7.** LPA Trajectories From 3-Class LPA Latent Trajectory Models With Varying Internal Model Structure: Male Participants **eFigure 8.** MVPA Trajectories From 3-Class MVPA Latent Trajectory Models With Varying Internal Model Structure: Male Participants **eFigure 9.** MVPA Trajectories From 3-Class MVPA Latent Trajectory Models With Varying Internal Model Structure: Male Participants **eFigure 10.** Observed MVPA and LPA Individual Trajectories by Most Likely Class From the Final 3-Class MVPA and LPA Trajectory Models **eFigure 11.** Physical Activity Trajectories From the Final 3-Class MVPA and LPA Latent Trajectory Models When Derived in the Maximum Sample Size (Not Restricted to Those With Complete Data on Confounders and Hip Outcomes) **eFigure 12.** MVPA/LPA Missing Data Patterns and Proportions **eFigure 13.** Causal Diagram Depicting the Assumptions of a Negative-Outcome Control Study to Evaluate the Association of Physical Activity Throughout Adolescence on Peak Hip Strength in Young Adults **eFigure 14.** Scatter Plot of Observed MVPA and LPA by Age and Sex **eFigure 15.** Association of MVPA and LPA Trajectory and Physical Activity Gravitational Impacts With Adult Leg Length (the Negative-Outcome Control) **eReferences.**
