# Supplementary material for: Physical Activity Throughout Adolescence and Peak Hip Strength in Young Adults
Source: JAMA Netw Open. 2020 Aug 17;3(8):e2013463. doi: 10.1001/jamanetworkopen.2020.13463 (PMC7431998; doi:10.1001/jamanetworkopen.2020.13463)
Supplement: Supplement. — eMethods. Latent Trajectory Modeling and Sensitivity Analysis for Uncontrolled Confounding eTable 1. Characteristics of the Initial Latent Trajectory Models With Varying Number of Classes eTable 2. Characteristics of the 3-Class Latent Trajectory Models With Varying Internal Model Structure eTable 3. Results of the Final 3-Class MVPA and LPA Latent Trajectory Models: Male Participants eTable 4. Results of the Final 3-Class MVPA and LPA Latent Trajectory Models: Female Participants eTable 5. Comparison Between Participants Included in Main Analysis With MVPA/LPA Accelerometer Assessments at All 4 Ages and Participants Included in Main Analysis With At Least 1 Missing MVPA/LPA Accelerometer Assessment eTable 6. Comparison Between Participants Included in Main Analysis and Participants Excluded From Main Analysis Due to Missing All 4 MVPA/LPA Accelerometer Assessments eTable 7. Association of Model Covariates With Adult Hip Strength Markers and Leg Length eTable 8. Characteristics of ALSPAC Participants Included in the Vertical Impact Analysis eTable 9. Associations of MVPA and LPA Trajectory With Hip Strength Markers: Male Participants eTable 10. Associations of MVPA and LPA Trajectory With Hip Strength Markers: Female Participants eTable 11. Association of Physical Activity Gravitational Impacts With Hip Strength Markers eFigure 1. Latent Trajectory Model Conceptualizations eFigure 2. MVPA Trajectories From Initial Latent Trajectory Models With Varying Number of Classes: Male Participants eFigure 3. LPA Trajectories From Initial Latent Trajectory Models With Varying Number of Classes: Male Participants eFigure 4. MVPA Trajectories From Initial Latent Trajectory Models With Varying Number of Classes: Female Participants eFigure 5. LPA Trajectories From Initial Latent Trajectory Models With Varying Number of Classes: Female Participants eFigure 6. MVPA Trajectories From 3-Class MVPA Latent Trajectory Models With Varying Internal Model Structure: Male Participants eFigu [file jamanetwopen-3-e2013463-s001.pdf]

Supplementary Online Content

Elhakeem A, Heron J, Tobias JH, Lawlor DA. Physical activity throughout adolescence and peak hip strength in young adults. *JAMA Netw Open*. 2020;3(8):e2013463. doi:10.1001/jamanetworkopen.2020.13463

eMethods. Latent Trajectory Modeling and Sensitivity Analysis for Uncontrolled Confounding

eTable 1. Characteristics of the Initial Latent Trajectory Models With Varying Number of Classes

eTable 2. Characteristics of the 3-Class Latent Trajectory Models With Varying Internal Model Structure

eTable 3. Results of the Final 3-Class MVPA and LPA Latent Trajectory Models: Male Participants

eTable 4. Results of the Final 3-Class MVPA and LPA Latent Trajectory Models: Female Participants

eTable 5. Comparison Between Participants Included in Main Analysis With MVPA/LPA Accelerometer Assessments at All 4 Ages and Participants Included in Main Analysis With At Least 1 Missing MVPA/LPA Accelerometer Assessment

eTable 6. Comparison Between Participants Included in Main Analysis and Participants Excluded From Main Analysis Due to Missing All 4 MVPA/LPA Accelerometer Assessments

eTable 7. Association of Model Covariates With Adult Hip Strength Markers and Leg Length

eTable 8. Characteristics of ALSPAC Participants Included in the Vertical Impact Analysis

eTable 9. Associations of MVPA and LPA Trajectory With Hip Strength Markers: Male Participants

eTable 10. Associations of MVPA and LPA Trajectory With Hip Strength Markers: Female Participants

eTable 11. Association of Physical Activity Gravitational Impacts With Hip Strength Markers

eFigure 1. Latent Trajectory Model Conceptualizations

eFigure 2. MVPA Trajectories From Initial Latent Trajectory Models With Varying Number of Classes: Male Participants

eFigure 3. LPA Trajectories From Initial Latent Trajectory Models With Varying Number of Classes: Male Participants

eFigure 4. MVPA Trajectories From Initial Latent Trajectory Models With Varying Number of Classes: Female Participants

eFigure 5. LPA Trajectories From Initial Latent Trajectory Models With Varying Number of Classes: Female Participants

eFigure 6. MVPA Trajectories From 3-Class MVPA Latent Trajectory Models With Varying Internal Model Structure: Male Participants

© 2020 Elhakeem A et al. *JAMA Network Open*.

eFigure 7. LPA Trajectories From 3-Class LPA Latent Trajectory Models With Varying Internal Model Structure: Male Participants

eFigure 8. MVPA Trajectories From 3-Class MVPA Latent Trajectory Models With Varying Internal Model Structure: Male Participants

eFigure 9. MVPA Trajectories From 3-Class MVPA Latent Trajectory Models With Varying Internal Model Structure: Male Participants

eFigure 10. Observed MVPA and LPA Individual Trajectories by Most Likely Class From the Final 3-Class MVPA and LPA Trajectory Models

eFigure 11. Physical Activity Trajectories From the Final 3-Class MVPA and LPA Latent Trajectory Models When Derived in the Maximum Sample Size (Not Restricted to Those With Complete Data on Confounders and Hip Outcomes)

eFigure 12. MVPA/LPA Missing Data Patterns and Proportions

eFigure 13. Causal Diagram Depicting the Assumptions of a Negative-Outcome Control Study to Evaluate the Association of Physical Activity Throughout Adolescence on Peak Hip Strength in Young Adults

eFigure 14. Scatter Plot of Observed MVPA and LPA by Age and Sex

eFigure 15. Association of MVPA and LPA Trajectory and Physical Activity Gravitational Impacts With Adult Leg Length (the Negative-Outcome Control)

eReferences

This supplementary material has been provided by the authors to give readers additional information about their work.

© 2020 Elhakeem A et al. *JAMA Network Open*.

eMethods. Latent Trajectory Modeling and Sensitivity Analysis for Uncontrolled Confounding

Latent trajectory modeling

Latent trajectory models were used to derive trajectory subgroups (latent classes) for time spent in MVPA and LPA from age 12 to 25 years (assessed at mean ages 12, 14, 16 and 25 years). These models aim to classify individuals into distinct subgroups that share similar trajectories such that individuals within a group are more similar than individuals between groups (1-8). Models were applied separately to males and females and to MVPA and LPA to derive trajectories for time spent in each intensity (eFigure 1). Random intercepts were used to allow variation in baseline activity (at age 12 years). Growth (i.e. change with age in time spent at MVPA and LPA) was captured by random linear and quadratic slopes. Factor loadings were fixed at the mean age at physical activity assessment.

In initial model parameterisations, the individual growth trajectories within classes were assumed to be homogenous by fixing the variance and covariance estimates for the growth factors within each class to zero. We used this model structure to identify the optimal number of classes by testing models in a stepwise fashion starting with a 1-class model (i.e. a model which assumes there are no subgroups and that all individuals follow the same trajectory over time) up to models with 6 latent trajectory classes. To aid model convergence, all the residual variances and variance-covariance matrix were fixed across latent classes. The class-specific mean trajectories from each of these models are shown in eFigure 2 and eFigure 3 for MVPA and LPA respectively in males and in eFigure 4 and eFigure 5 for MVPA and LPA respectively in females.

The models with the favoured number of subgroups (latent classes) were chosen based on a combination of theory, interpretability, meaningfulness and fit indices. Interpretability and meaningfulness were informed by the smallest class size whereby we disfavoured classes with too few participants. Fit indices included the Bayesian Information Criterion (BIC), sample-size adjusted BIC, Vuong-Lo-Mendell-Rubin test (VLMR) and entropy. Where model indices were in conflict with each other, we selected the model with the lower number of classes that was still theoretically meaningful. The characteristics of the initial models with varying number of classes are presented in eTable 1. Following this process, we selected 3-class MVPA models (i.e. trajectory subgroups) and 3-class LPA models for both males and females.

© 2020 Elhakeem A et al. *JAMA Network Open*.

After identifying the optimal number of classes, we considered alternative specifications of within-class heterogeneity. We tested models that allowed for within class heterogeneity in the intercept, followed by models allowing variation in individual linear slopes and finally models that allow within class heterogeneity in both the intercept and linear slope. The class-specific mean trajectories from each of these 3-class models with varying model structures are shown in eFigure 6 and eFigure 7 for MVPA and LPA respectively in males and in eFigure 8 and eFigure 9 for MVPA and LPA respectively in females. The characteristics of each of these 3-class models with varying model structures are presented in eTable 2.

The final models were selected based on the lowest BIC or the highest entropy where two models had similar BIC. Model selection was based on a trade-off between efficiency and validation, with the aim of summarising distinct trajectories of the data as parsimonious as possible and not just the maximisation of model fits. For MVPA, we favoured models that allowed for within class variance in the intercept and linear slope in males and models without within class heterogeneity in females (eTable 2). For LPA in both males and females, we favoured models that allowed for within class variance in the linear slope (eTable 2).

The results of the final favoured MVPA and LPA models in terms of how they relate to measured variables are shown in eTable 3 for males and eTable 4 for females. eFigure 10 shows the observed MVPA and LPA individual trajectories colour-coded by most likely class from the final favoured MVPA and LPA models. This figure helps visualise the extent to which individual variability is explained by the latent group trajectory as well as the extent of overlap between observations of individuals from different groups. The figures show that there was good separation between trajectory classes for both MVPA and LPA. To check the validity of the MVPA trajectory subgroups in females, and in particular the *Low Adolescent-High Adult MVPA* group, we compared how adult i.e. age 25 BMI and fat mass as well as change in these between age 16 and age 25 vary between subgroups. The results showed that, as expected, the 8% of females in the *Low Adolescent-High Adult MVPA* group had lower BMI and fat mass at age 25 as well as slower gains in BMI and fat mass between ages 16 and 25 than the other 2 groups. This provides further support for the validity of these sub-groupings.

Latent trajectory models were estimated using the expectation-maximisation (EM)-algorithm in Mplus version 8 (Muthen & Muthen). The Mplus code used to fit the models is available at https://github.com/aelhak/PA-Hip-BMD. To investigate and avoid multiple local solutions,

© 2020 Elhakeem A et al. *JAMA Network Open.*

we specified the number of initial stage random sets of starting values to generate, and the number of final stage optimizations to use. Specifically, 3500 random sets of starting values for the initial stage and 350 final stage optimizations, along with 25 initial stage iterations were used. We fitted all the models to the analytic sample i.e. those with at least 1 MVPA and LPA measure from any one age in addition to complete data on all confounders and adult hip outcomes. To check stability of the identified groups we repeated models in the maximal sample. eFigure 11 shows that the favoured models resulted in very similar trajectories when they were re-estimated in the maximal sample sizes.

Latent trajectory models handle missing physical activity data using full information maximum likelihood estimation, which allows inclusion of all participants with at least 1 MVPA and LPA measure under the missing at random assumption. eFigure 12 shows the MVPA and LPA missing data patterns and proportions. eTable 5 shows that among those included in the latent trajectory models, the probability of missing accelerometer data was related to the confounders in the subsequent regression models, indicating the data may be consistent with missing at random. eTable 6 shows that those missing all four accelerometer assessments, who were as a result excluded from the study analysis, were socioeconomically different from the analytical sample, which might limit the generalisability of the associations of MVPA and LPA with adult hip outcomes. Participants with missing data on covariates (17.8% of those potentially eligible) were excluded, which might introduce a bias if they had systematically different adult hip bone density and geometrical measurements.

Sensitivity analysis for uncontrolled confounding

Because the observational associations between adolescent physical activity and adult hip strength may be biased by uncontrolled confounding, we used a negative outcome control study to explore the possibility that our results were biased by uncontrolled confounding (eFigure 13). A negative control study is one that includes the same participants as the 'real' study but examines the association of the 'real' exposure with a negative control outcome (or vice versa) (9, 10). Under the assumptions that the negative control outcome (or exposure) has been correctly selected such that confounders (measured or unmeasured) relate similarly to it as the 'real' outcome (or exposure), and that there is no plausible causal link between the 'real' exposure and negative control outcome, a similar (non-null) association in the negative control outcome would suggest that the main (hip strength) result may be explained by

uncontrolled confounding (9 10). Negative control studies have increasingly been used to explore bias due to uncontrolled confounding in observational studies (9-12).

We used adult leg length from age 25 years as a negative control outcome. Leg length was measured in centimetres and was calculated by subtracting seated height (measured using a Harpenden sitting height table and anthropometer board) from standing height (measured using a Harpenden wall-mounted stadiometer). Leg length was selected as negative control outcome because it is known to be sensitive to early life environment (13-15). This is supported by evidence that legs grow rapidly between infancy and puberty with most of the growth being completed by puberty (16, 17), therefore factors that influence leg growth are largely occurring in infancy and early childhood. This means that adult leg length therefore likely shares similar measured and unmeasured (including genetic) determinants as adult hip strength (Figure 1). Because we considered it implausible that leg length would be influenced by physical activity through adolescence (intensity or impact) therefore, any association with between adolescent physical activity and adult leg length would be likely due to confounding and suggest the same may be true for our main (real i.e. hip strength) analyses (9, 10).

For the study, the main (i.e. adult hip strength) analysis and the negative control analyses (i.e. adult leg length) were performed on the same sample (i.e. n=981 males and n=1588 females for the MVPA and LPA analyses, and n=478 for the vertical impacts analyses) Table 7 shows that overall, the measured early life study confounders were associated with both adult hip strength and adult leg length.

eTable 1. Characteristics of the Initial Latent Trajectory Models With Varying Number of Classes

Number of classes (k)	Parameters	BIC	adjusted BIC	VLMR	Entropy	Sample size per class
**MVPA models – males**						
k=1	4	22161.561	22148.857	-	-	981
k=2	8	22012.839	21987.431	P=0.0531	0.719	857/124
**k=3**	**12**	**21960.638**	**21922.525**	**P=0.0108**	**0.582**	**787/126/68**
k=4	16	21935.360	21884.544	P=0.1769	0.646	750/120/94/17
k=5	20	21938.177	21874.657	P=0.1971	0.635	758/99/97/14/13
k=6	24	21944.876	21868.652	P=0.2288	0.577	677/177/48/40/27/12
**LPA models – males**						
k=1	4	25730.914	25718.210	–	–	981
k=2	8	25555.923	25530.514	P<0.0001	0.467	643/338
**k=3**	**12**	**25515.531**	**25477.419**	**P=0.5774**	**0.581**	**713/194/74**
k=4	16	25506.636	25455.819	P=0.0502	0.637	735/151/86/9
k=5	20	25520.882	25457.362	P=0.2699	0.583	703/143/114/13/8
k=6	24	25531.801	25455.576	P=0.3740	0.598	655/152/130/17/14/12
**MVPA models – females**						
k=1	4	34529.173	34516.466	-	-	1588
k=2	8	34255.061	34229.646	P=0.0009	0.648	1352/236
**k=3**	**12**	**34144.151**	**34106.029**	**P=0.0002**	**0.603**	**1301/252/35**
k=4	16	34082.374	34031.546	P=0.0672	0.655	1233/311/33/11
k=5	20	34049.617	33986.081	P=0.6344	0.642	1093/424/39/19/13
k=6	24	34024.889	33948.646	P=0.5023	0.673	1060/453/40/21/13/1
**LPA models – females**						
k=1	4	42224.391	42211.684	-	-	1588
k=2	8	41849.572	41824.157	P<0.0001	0.538	1065/523
**k=3**	**12**	**41834.791**	**41796.669**	**P=0.3804**	**0.545**	**846/661/81**
k=4	16	41830.518	41779.690	P=0.4496	0.485	782/590/150/66
k=5	20	41834.052	41770.516	P=0.3770	0.561	1012/354/130/71/21
k=6	24	41835.625	41759.382	P=0.2940	0.605	998/344/137/83/22/4

© 2020 Elhakeem A et al. *JAMA Network Open*.

eTable 2. Characteristics of the 3-Class Latent Trajectory Models With Varying Internal Model Structure

Model	Parameters	BIC	adjusted BIC	Entropy	Sample size per class
**3-class MVPA models – males**					
Model A	12	21960.638	21922.525	0.582	787/126/68
Model B	13	21955.226	21913.938	0.568	818/117/46
Model C	13	21954.222	21912.934	0.740	794/177/10
**Model D**	**14**	**21952.494**	**21908.030**	**0.767**	**880/52/49**
**3-class LPA models – males**					
Model A	12	25515.531	25477.419	0.581	713/194/74
Model B	13	25490.728	25449.440	0.486	510/465/6
**Model C**	**13**	**25519.558**	**25478.270**	**0.578**	**740/123/118**
Model D	14	25489.900	25445.436	0.645	939/38/4
**3-class MVPA models – females**					
**Model A**	**12**	**34144.151**	**34106.029**	**0.603**	**1301/252/35**
Model B	13	34081.214	34039.916	0.756	1541/32/15
Model C	13	34085.624	34044.326	0.753	1278/298/12
Model D	14	34075.544	34031.069	0.765	1367/208/13
**3-class LPA models – females**					
Model A	12	41834.791	41796.669	0.545	846/661/81
Model B	13	41805.837	41764.538	0.516	1151/385/52
**Model C**	**13**	**41840.103**	**41798.805**	**0.570**	**825/701/62**
Model D	14	41795.823	41751.348	0.441	1193/336/59

Model A: no within class heterogeneity
Model B: within class variance in the intercept
Model C: within class variance in the linear slope
Model D: within class variance in the intercept and linear slope

eTable 3. Results of the Final 3-Class MVPA and LPA Latent Trajectory Models: Male Participants

	MVPA trajectories				LPA trajectories		
	High Early-Adolescent MVPA	High Mid-Adolescent MVPA	Low Adolescent MVPA		High Decreasing LPA	Moderate Decreasing LPA	Low Non-Linear LPA
MVPA and LPA at each age [mean (SD)]							
MVPA age 12	128.6 (20.1)	96.2 (25.1)	59.5 (22.7)		70.6 (25.0)	65.7 (28.7)	56.3 (28.8)
MVPA age 14	78.0 (18.2)	116.1 (23.5)	53.7 (23.5)		68.1 28.0)	58.7 (28.0)	47.7 (25.6)
MVPA age 16	55.8 (30.6)	117.8 (35.4)	50.7 (25.1)		64.9 (38.3)	55.4 (27.9)	41.6 (31.2)
MVPA age 25	42.6 (21.4)	43.3 (24.7)	55.8 (33.9)		57.7 (31.7)	54.2 (33.7)	49.8 (30.8)
LPA age 12	382.6 (48.3)	377.4 (48.9)	364.6 (62.2)		452.9 (49.3)	364.5 (43.8)	289.2 (49.6)
LPA age 14	351.8 (62.7)	350.1 (57.1)	324.7 (63.3)		404.4 (43.6)	327.6 (45.8)	229.0 (43.8)
LPA age 16	287.7 (82.3)	330.0 (74.0)	282.9 (65.8)		382.2 (50.2)	283.5 (50.8)	200.4 (39.8)
LPA age 25	131.9 (47.5)	149.4 (42.6)	149.4 (62.6)		206.2 (85.0)	141.3 (49.5)	123.4 (48.0)
Fat mass index age 10y – kg/m$^{1.2}$ [mean (SD)]	3.0 (1.6)	3.8 (2.3)	5.0 (3.1)		4.5 (2.6)	4.8 (3.0)	5.4 (3.2)
Lean mass index age 10y – kg/m$^{1.2}$ [mean (SD)]	17.3 (1.3)	17.1 (1.1)	16.9 (1.3)		16.9 (1.3)	17.0 (1.3)	17.0 (1.2)
Height at age 10y – cm [mean (SD)]	139.4 (5.1)	140.3 (6.1)	140.2 (6.1)		139.3 (5.9)	140.1 (6.1)	141.4 (5.7)
Ethnicity [No. (%)]							
White	48 (98)	52 (100)	863 (98)		122 (99)	727 (98)	114 (97)
Non-white	1 (2)	0 (0)	17 (2)		1 (1)	13 (2)	4 (3)
Maternal education [No. (%)]							
Degree or higher	10 (20)	11 (21)	212 (25)		21 (17)	170 (23)	42 (36)
Lower than degree	39 (80)	41 (79)	668 (76)		102 (83)	570 (77)	76 (64)

© 2020 Elhakeem A et al. *JAMA Network Open*.

eTable 4. Results of the Final 3-Class MVPA and LPA Latent Trajectory Models: Female Participants

	MVPA trajectories				LPA trajectories		
	High Adolescent MVPA	Low Adolescent-High Adult MVPA	Low Adolescent-Low Adult MVPA		High Decreasing LPA	Moderate Decreasing LPA	Low Non-Linear LPA
					471.4 (43.6)	396.9 (42.0)	325.9 (43.8)
LPA age 12	384.8 (56.9)	344.7 (54.2)	359.3 (59.1)		415.0 (37.4)	342.9 (40.6)	267.7 (43.2)
LPA age 14	329.0 (60.2)	289.5 (41.9)	304.3 (59.6)		384.4 (55.1)	303.1 (45.0)	228.2 (41.3)
LPA age 16	289.2 (57.2)	266.2 (46.7)	265.1 (63.3)		213.3 (67.8)	156.7 (54.9)	135.0 (44.3)
LPA age 25	159.3 (58.3)	162.8 (61.0)	144.5 (51.0)				
MVPA age 12	70.6 (20.3)	42.6 (20.4)	40.6 (15.6)		57.0 (20.1)	48.9 (20.0)	41.6 (18.7)
MVPA age 14	72.4 (23.4)	38.0 (17.5)	37.6 (16.8)		52.3 (25.3)	47.4 (22.5)	39.1 (21.0)
MVPA age 16	68.2 (22.2)	36.7 (14.0)	32.8 (15.8)		45.3 (27.8)	41.3 (21.0)	35.7 (20.7)
MVPA age 25	58.5 (22.0)	105.7 (21.5)	37.1 (17.7)		48.4 (25.6)	46.9 (26.0)	45.9 (28.2)
Fat mass index age 10y – kg/m$^{1.2}$ [mean (SD)]	5.7 (2.9)	6.1 (3.2)	6.3 (3.1)		5.4 (3.1)	5.8 (2.8)	6.6 (3.2)
Lean mass index age 10y – kg/m$^{1.2}$ [mean (SD)]	15.9 (1.3)	15.7 (1.4)	15.7 (1.4)		15.6 (1.2)	15.7 (1.4)	15.8 (1.4)
Height at age 10y – cm [mean (SD)]	138.8 (6.5)	139.5 (6.4)	138.9 (6.3)		136.5 (6.9)	138.3 (6.2)	139.6 (6.2)
Ethnicity [No. (%)]							
White	249 (99)	35 (100)	1270 (98)		62 (100)	688 (98)	804 (97)
Non-white	3 (1)	0 (0)	31 (2)		0 (0)	13 (2)	21 (3)
Maternal education [No. (%)]							
Degree or higher	67 (27)	16 (46)	247 (19)		9 (15)	123 (18)	198 (24)
Lower than degree	185 (73)	19 (54)	1054 (81)		53 (85)	578 (82)	627 (76)

© 2020 Elhakeem A et al. *JAMA Network Open*.

eTable 5. Comparison Between Participants Included in Main Analysis With MVPA/LPA Accelerometer Assessments at All 4 Ages and Participants Included in Main Analysis With At Least 1 Missing MVPA/LPA Accelerometer Assessment

	Males			Females	
	Included in main analysis: four accelerometer assessments available (n=103)	Included in main analysis: missing ≥ 1 accelerometer assessment (n=878)		Included in main analysis: four accelerometer assessments available (n=191)	Included in main analysis: missing ≥ 1 accelerometer assessment (n=1397)
Fat mass index age 10y – $kg/m^{1.2}$ [mean (SD)]	4.6 (2.5)	4.9 (3.1)		6.0 (2.9)	6.3 (3.1)
Lean mass index age 10y – $kg/m^{1.2}$ [mean (SD)]	16.7 (1.3)	17.0 (1.3)		15.7 (1.4)	15.7 (1.4)
Height at age 10y – cm [mean (SD)]	140.1 (6.0)	140.1 (6.0)		138.1 (5.8)	139.0 (6.3)
Ethnicity [No. (%)]					
White	99 (96)	864 (98)		191 (100)	1363 (98)
Non-white	4 (4)	14 (2)		0 (0)	34 (2)
Maternal education [No. (%)]					
Degree or higher	23 (22)	210 (24)		40 (21)	290 (21)
Lower than degree	80 (78)	668 (76)		151 (79)	1107 (79)

© 2020 Elhakeem A et al. *JAMA Network Open*.

eTable 6. Comparison Between Participants Included in Main Analysis and Participants Excluded From Main Analysis Due to Missing All 4 MVPA/LPA Accelerometer Assessments

	Males			Females	
	Included in main analysis (n=981)	Excluded due to missing data (n=4405)		Included in main analysis (n=1588)	Excluded due to missing data (n=3673)
Fat mass index age 10y – kg/m$^{1.2}$ [mean (SD)]	4.8 (3.0)	5.0 (3.3)		6.2 (3.0)	6.7 (3.4)
Lean mass index age 10y – kg/m$^{1.2}$ [mean (SD)]	17.0 (1.3)	17.2 (1.4)		15.7 (1.4)	16.0 (1.5)
Height at age 10y – cm [mean (SD)]	140.1 (6.0)	139.9 (6.1)		138.9 (6.3)	140.2 (6.6)
Ethnicity [No. (%)]					
White	963 (98)	2670 (97)		1554 (98)	2670 (97)
Non-white	18 (2)	93 (3)		34 (2)	93 (3)
Maternal education [No. (%)]					
Degree or higher	233 (24)	257 (9)		330 (21)	2540 (91)
Lower than degree	748 (76)	2540 (91)		1258 (79)	257 (9)

© 2020 Elhakeem A et al. *JAMA Network Open*.

eTable 7. Association of Model Covariates With Adult Hip Strength Markers and Leg Length

	Males	Females
THBMD		
Fat mass index at 10y	0.05 (-0.01 to 0.11)	0.19 (0.14 to 0.24)
Lean mass index at 10y	0.29 (0.23 to 0.35)	0.28 (0.23 to 0.33)
Height at 10y	0.14 (0.08 to 0.20)	0.21 (0.16 to 0.25)
Race		
White	1.00 (reference)	1.00 (reference)
Non-white	0.14 (-0.33 to 0.60)	0.23 (-0.11 to 0.57)
Maternal education		
Degree or higher	1.00 (reference)	1.00 (reference)
Lower than degree	0.02 (-0.12 to 0.17)	-0.10 (-0.22 to 0.03)
FNBMD		
Fat mass index at 10y	0.10 (0.04 to 0.16)	0.21 (0.16 to 0.25)
Lean mass index at 10y	0.30 (0.25 to 0.36)	0.30 (0.25 to 0.34)
Height at 10y	0.20 (0.13 to 0.26)	0.27 (0.22 to 0.31)
Race		
White	1.00 (reference)	1.00 (reference)
Non-white	0.27 (-0.20 to 0.74)	0.02 (-0.32 to 0.36)
Maternal education		
Degree or higher	1.00 (reference)	1.00 (reference)
Lower than degree	0.05 (-0.10 to 0.19)	-0.07 (-0.19 to 0.05)
Leg length		
Fat mass index at 10y	0.02 (-0.04 to 0.08)	0.05 (0.00 to 0.09)
Lean mass index at 10y	0.21 (0.15 to 0.27)	0.18 (0.13 to 0.22)
Height at 10y	0.63 (0.58 to 0.68)	0.61 (0.57 to 0.65)
Race		
White	1.00 (reference)	1.00 (reference)
Non-white	-0.28 (-0.75 to 0.19)	-0.12 (-0.46 to 0.22)
Maternal education		
Degree or higher	1.00 (reference)	1.00 (reference)
Lower than degree	-0.14 (-0.29 to 0.01)	-0.20 (-0.32 to -0.08)

Estimates from linear regression models, with all outcomes entered in SD units.

© 2020 Elhakeem A et al. *JAMA Network Open*.

eTable 8. Characteristics of ALSPAC Participants Included in the Vertical Impact Analysis

	Males (n=183)	Females (n=295)
Age at Newtest monitor assessment – years [mean (SD)]	17.7 (0.3)	17.7 (0.3)
Number of impacts according to g-band [median (IQR)]		
0.5 up to 1.1 g	21049 (11093, 36008)	16308 (9828, 27291)
1.1 up to 3.1 g	4446 (2482, 9319)	3676 (1987, 6857)
3.1 up to 5.1 g	217 (112, 463)	186 (72, 419)
>5.1 g	71 (34, 175)	50 (21, 106)
Hip strength markers at age 25y [mean (SD)]		
Total hip BMD – *g/cm²*	1.14 (0.2)	1.07 (0.1)
Femur neck BMD – *g/cm²*	1.12 (0.2)	1.06 (0.1)
Femur neck width – *mm*	33.8 (2.6)	28.8 (2.1)
Cross-sectional area – *mm²*	186.6 (31.2)	153.3 (21.8)
Section modulus – *mm³*	909.6 (189.8)	645.5 (125.3)
Cross-sectional moment of inertia – *mm⁴*	16274 (4200)	9716 (2380)
Negative control outcome: leg length (cm) at age 25y [mean (SD)]	85.8 (4.7)	77.7 (3.9)
Fat mass index at age 10y – $kg/m^{1.2}$ *[mean (SD)]*	4.9 (3.0)	6.3 (3.1)
Lean mass index age 10y – $kg/m^{1.2}$ *[mean (SD)]*	16.8 (1.4)	15.9 (1.5)
Height at age 10y – *cm* [mean (SD)]	140.7 (6.2)	138.9 (6.0)
Maternal education [No. (%)]		
Degree or higher	50 (27)	71 (24)
Lower than degree	133 (73)	224 (76)
Race [No. (%)]		
White	179 (98)	288 (98)
Non-white	4 (2)	7 (2)

© 2020 Elhakeem A et al. *JAMA Network Open*.

eTable 9. Associations of MVPA and LPA Trajectory With Hip Strength Markers: Male Participants

	Model 1	Model 2
**MVPA trajectory and hip strength**		
THBMD		
Low Adolescent MVPA	1 (reference group)	1 (reference group)
High Early-Adolescent MVPA	0.53 (0.24 to 0.82)	0.43 (0.15 to 0.71)
High Mid-Adolescent MVPA	0.41 (0.13 to 0.69)	0.35 (0.09 to 0.62)
FNBMD		
Low Adolescent MVPA	1 (reference group)	1 (reference group)
High Early-Adolescent MVPA	0.45 (0.16 to 0.74)	0.38 (0.11 to 0.66)
High Mid-Adolescent MVPA	0.38 (0.10 to 0.66)	0.33 (0.07 to 0.60)
MNW		
Low Adolescent MVPA	1 (reference group)	1 (reference group)
High Early-Adolescent MVPA	0.40 (0.11 to 0.69)	0.30 (0.04 to 0.55)
High Mid-Adolescent MVPA	0.42 (0.14 to 0.70)	0.34 (0.09 to 0.59)
CSA		
Low Adolescent MVPA	1 (reference group)	1 (reference group)
High Early-Adolescent MVPA	0.54 (0.25 to 0.82)	0.44 (0.18 to 0.70)
High Mid-Adolescent MVPA	0.45 (0.17 to 0.73)	0.39 (0.14 to 0.63)
SM		
Low Adolescent MVPA	1 (reference group)	1 (reference group)
High Early-Adolescent MVPA	0.50 (0.21 to 0.78)	0.37 (0.12 to 0.62)
High Mid-Adolescent MVPA	0.49 (0.22 to 0.77)	0.41 (0.16 to 0.65)
CSMI		
Low Adolescent MVPA	1 (reference group)	1 (reference group)
High Early-Adolescent MVPA	0.51 (0.22 to 0.79)	0.38 (0.13 to 0.62)
High Mid-Adolescent MVPA	0.51 (0.23 to 0.78)	0.42 (0.18 to 0.65)
**LPA trajectory and hip strength**		
THBMD		
Low Non-Linear LPA	1 (reference group)	1 (reference group)
Moderate Decreasing LPA	0.08 (-0.12 to 0.27)	0.06 (-0.13 to 0.24)
High Decreasing LPA	0.09 (-0.16 to 0.34)	0.08 (-0.16 to 0.33)
FNBMD		
Low Non-Linear LPA	1 (reference group)	1 (reference group)
Moderate Decreasing LPA	0.15 (-0.04 to 0.34)	0.12 (-0.06 to 0.30)
High Decreasing LPA	0.19 (-0.06 to 0.45)	0.16 (-0.08 to 0.40)
MNW		
Low Non-Linear LPA	1 (reference group)	1 (reference group)
Moderate Decreasing LPA	0.07 (-0.12 to 0.26)	0.02 (-0.15 to 0.19)
High Decreasing LPA	-0.02 (-0.27 to 0.24)	-0.12 (-0.34 to 0.11)
CSA		
Low Non-Linear LPA	1 (reference group)	1 (reference group)
Moderate Decreasing LPA	0.15 (-0.04 to 0.34)	0.11 (-0.06 to 0.27)
High Decreasing LPA	0.15 (-0.11 to 0.40)	0.07 (-0.15 to 0.30)
SM		
Low Non-Linear LPA	1 (reference group)	1 (reference group)
Moderate Decreasing LPA	0.09 (-0.10 to 0.29)	0.05 (-0.12 to 0.22)
High Decreasing LPA	0.05 (-0.20 to 0.30)	-0.02 (-0.25 to 0.20)
CSMI		
Low Non-Linear LPA	1 (reference group)	1 (reference group)

© 2020 Elhakeem A et al. *JAMA Network Open*.

Moderate Decreasing LPA	0.10 (-0.09 to 0.29)	0.04 (-0.12 to 0.21)
High Decreasing LPA	0.05 (-0.21 to 0.30)	-0.04 (-0.26 to 0.17)

Model 1 shows unadjusted estimates. Models 2 shows estimates adjusted for ethnicity, maternal education, child height, fat and lean mass indices, and age at hip scan. MVPA: moderate to-vigorous intensity physical activity. LPA: light intensity physical activity. THBMD: total hip bone mineral density, FNBMD: femur neck hip bone mineral density. MNW: minimum femur neck width. CSA: hip cross-sectional area. CSMI: hip cross-sectional moment of inertia. SM: hip section modulus.

© 2020 Elhakeem A et al. *JAMA Network Open*.

eTable 10. Associations of MVPA and LPA Trajectory With Hip Strength Markers: Female Participants

	Model 1	Model 2
**MVPA trajectory and hip strength**		
THBMD		
Low Adolescent-Low Adult MVPA	1 (reference group)	1 (reference group)
Low Adolescent-High Adult MVPA	-0.22 (-0.56 to 0.11)	-0.25 (-0.57 to 0.07)
High Adolescent MVPA	0.27 (0.13 to 0.40)	0.25 (0.12 to 0.38)
FNBMD		
Low Adolescent-Low Adult MVPA	1 (reference group)	1 (reference group)
Low Adolescent-High Adult MVPA	-0.08 (-0.41 to 0.26)	-0.12 (-0.44 to 0.20)
High Adolescent MVPA	0.29 (0.15 to 0.42)	0.28 (0.15 to 0.41)
MNW		
Low Adolescent-Low Adult MVPA	1 (reference group)	1 (reference group)
Low Adolescent-High Adult MVPA	0.22 (-0.11 to 0.56)	0.22 (-0.09 to 0.52)
High Adolescent MVPA	0.20 (0.06 to 0.33)	0.19 (0.06 to 0.31)
CSA		
Low Adolescent-Low Adult MVPA	1 (reference group)	1 (reference group)
Low Adolescent-High Adult MVPA	0.02 (-0.31 to 0.36)	-0.02 (-0.31 to 0.28)
High Adolescent MVPA	0.33 (0.20 to 0.47)	0.32 (0.20 to 0.44)
SM		
Low Adolescent-Low Adult MVPA	1 (reference group)	1 (reference group)
Low Adolescent-High Adult MVPA	0.09 (-0.25 to 0.42)	0.06 (-0.23 to 0.35)
High Adolescent MVPA	0.34 (0.20 to 0.47)	0.32 (0.20 to 0.44)
CSMI		
Low Adolescent-Low Adult MVPA	1 (reference group)	1 (reference group)
Low Adolescent-High Adult MVPA	0.16 (-0.17 to 0.50)	0.15 (-0.14 to 0.43)
High Adolescent MVPA	0.31 (0.18 to 0.44)	0.29 (0.18 to 0.41)
**LPA trajectory and hip strength**		
THBMD		
Low Non-Linear LPA	1 (reference group)	1 (reference group)
Moderate Decreasing LPA	0.05 (-0.05 to 0.15)	0.12 (0.02 to 0.22)
High Decreasing LPA	0.02 (-0.24 to 0.28)	0.14 (-0.11 to 0.39)
FNBMD		
Low Non-Linear LPA	1 (reference group)	1 (reference group)
Moderate Decreasing LPA	0.05 (-0.05 to 0.15)	0.13 (0.04 to 0.23)
High Decreasing LPA	0.05 (-0.20 to 0.31)	0.20 (-0.05 to 0.44)
MNW		
Low Non-Linear LPA	1 (reference group)	1 (reference group)
Moderate Decreasing LPA	-0.03 (-0.13 to 0.07)	0.06 (-0.03 to 0.15)
High Decreasing LPA	-0.13 (-0.39 to 0.12)	0.05 (-0.18 to 0.28)
CSA		
Low Non-Linear LPA	1 (reference group)	1 (reference group)
Moderate Decreasing LPA	0.03 (-0.07 to 0.13)	0.15 (0.06 to 0.24)
High Decreasing LPA	-0.01 (-0.27 to 0.25)	0.20 (-0.02 to 0.43)
SM		
Low Non-Linear LPA	1 (reference group)	1 (reference group)
Moderate Decreasing LPA	0.03 (-0.07 to 0.13)	0.15 (0.06 to 0.23)
High Decreasing LPA	-0.01 (-0.27 to 0.25)	0.20 (-0.02 to 0.42)
CSMI		
Low Non-Linear LPA	1 (reference group)	1 (reference group)
Moderate Decreasing LPA	0.01 (-0.09 to 0.11)	0.13 (0.04 to 0.21)
High Decreasing LPA	-0.06 (-0.32 to 0.20)	0.16 (-0.06 to 0.38)

Model 1 shows unadjusted estimates. Models 2 shows estimates adjusted for ethnicity, maternal education, child height, fat and lean mass indices, and age at hip scan. MVPA: moderate to-vigorous intensity physical activity.

LPA: light intensity physical activity. THBMD: total hip bone mineral density, FNBMD: femur neck hip bone mineral density. MNW: minimum femur neck width. CSA: hip cross-sectional area. CSMI: hip cross-sectional moment of inertia. SM: hip section modulus.

© 2020 Elhakeem A et al. *JAMA Network Open*.

eTable 11. Association of Physical Activity Gravitational Impacts With Hip Strength Markers

Impact g-band (per doubling in number of impacts)	Model 1	Model 2
THBMD		
0.5 up to 1.1 g	0.06 (-0.04 to 0.16)	0.06 (-0.03 to 0.16)
1.1 up to 3.1 g	0.03 (-0.06 to 0.12)	0.03 (-0.06 to 0.12)
3.1 up to 5.1 g	0.07 (-0.01 to 0.14)	0.06 (-0.02 to 0.13)
>5.1 g	0.10 (0.02 to 0.17)	0.09 (0.01 to 0.16)
FNBMD		
0.5 up to 1.1 g	0.01 (-0.08 to 0.11)	0.03 (-0.06 to 0.13)
1.1 up to 3.1 g	-0.01 (-0.10 to 0.08)	0.01 (-0.08 to 0.10)
3.1 up to 5.1 g	0.05 (-0.03 to 0.12)	0.05 (-0.03 to 0.12)
>5.1 g	0.08 (0.01 to 0.16)	0.08 (0.01 to 0.16)
MNW		
0.5 up to 1.1 g	0.02 (-0.08 to 0.11)	0.06 (-0.03 to 0.14)
1.1 up to 3.1 g	-0.03 (-0.12 to 0.05)	-0.01 (-0.08 to 0.07)
3.1 up to 5.1 g	0.00 (-0.07 to 0.07)	0.00 (-0.06 to 0.06)
>5.1 g	0.03 (-0.04 to 0.10)	0.03 (-0.04 to 0.09)
CSA		
0.5 up to 1.1 g	0.01 (-0.08 to 0.11)	0.05 (-0.04 to 0.14)
1.1 up to 3.1 g	-0.03 (-0.12 to 0.06)	-0.01 (-0.09 to 0.07)
3.1 up to 5.1 g	0.02 (-0.05 to 0.10)	0.02 (-0.05 to 0.09)
>5.1 g	0.07 (-0.01 to 0.14)	0.06 (-0.01 to 0.13)
SM		
0.5 up to 1.1 g	0.02 (-0.08 to 0.11)	0.06 (-0.03 to 0.14)
1.1 up to 3.1 g	-0.04 (-0.13 to 0.05)	-0.02 (-0.10 to 0.06)
3.1 up to 5.1 g	0.01 (-0.06 to 0.08)	0.00 (-0.06 to 0.07)
>5.1 g	0.05 (-0.02 to 0.13)	0.05 (-0.02 to 0.11)
CSMI		
0.5 up to 1.1 g	0.02 (-0.08 to 0.11)	0.05 (-0.04 to 0.14)
1.1 up to 3.1 g	-0.04 (-0.13 to 0.05)	-0.01 (-0.09 to 0.07)
3.1 up to 5.1 g	0.01 (-0.06 to 0.08)	0.02 (-0.05 to 0.09)
>5.1 g	0.05 (-0.02 to 0.13)	0.05 (-0.02 to 0.11)

Model 1 shows unadjusted estimates. Models 2 shows estimates adjusted for sex, ethnicity, maternal education, child height, fat and lean mass indices, and age at hip scan. THBMD: total hip bone mineral density, FNBMD: femur neck hip bone mineral density. MNW: minimum femur neck width. CSA: hip cross-sectional area. CSMI: hip cross-sectional moment of inertia. SM: hip section modulus.

© 2020 Elhakeem A et al. *JAMA Network Open*.

eFigure 1. Latent Trajectory Model Conceptualizations

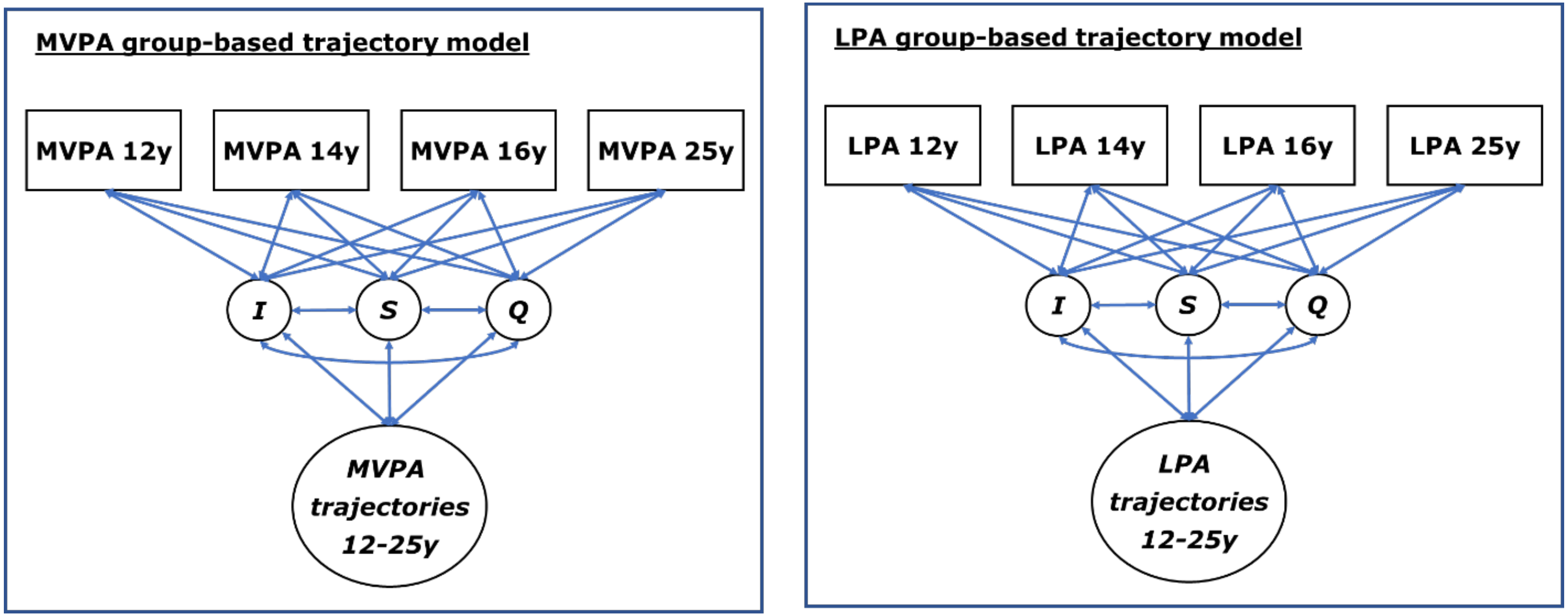

© 2020 Elhakeem A et al. *JAMA Network Open*.

eFigure 2. MVPA Trajectories From Initial Latent Trajectory Models With Varying Number of Classes: Male Participants

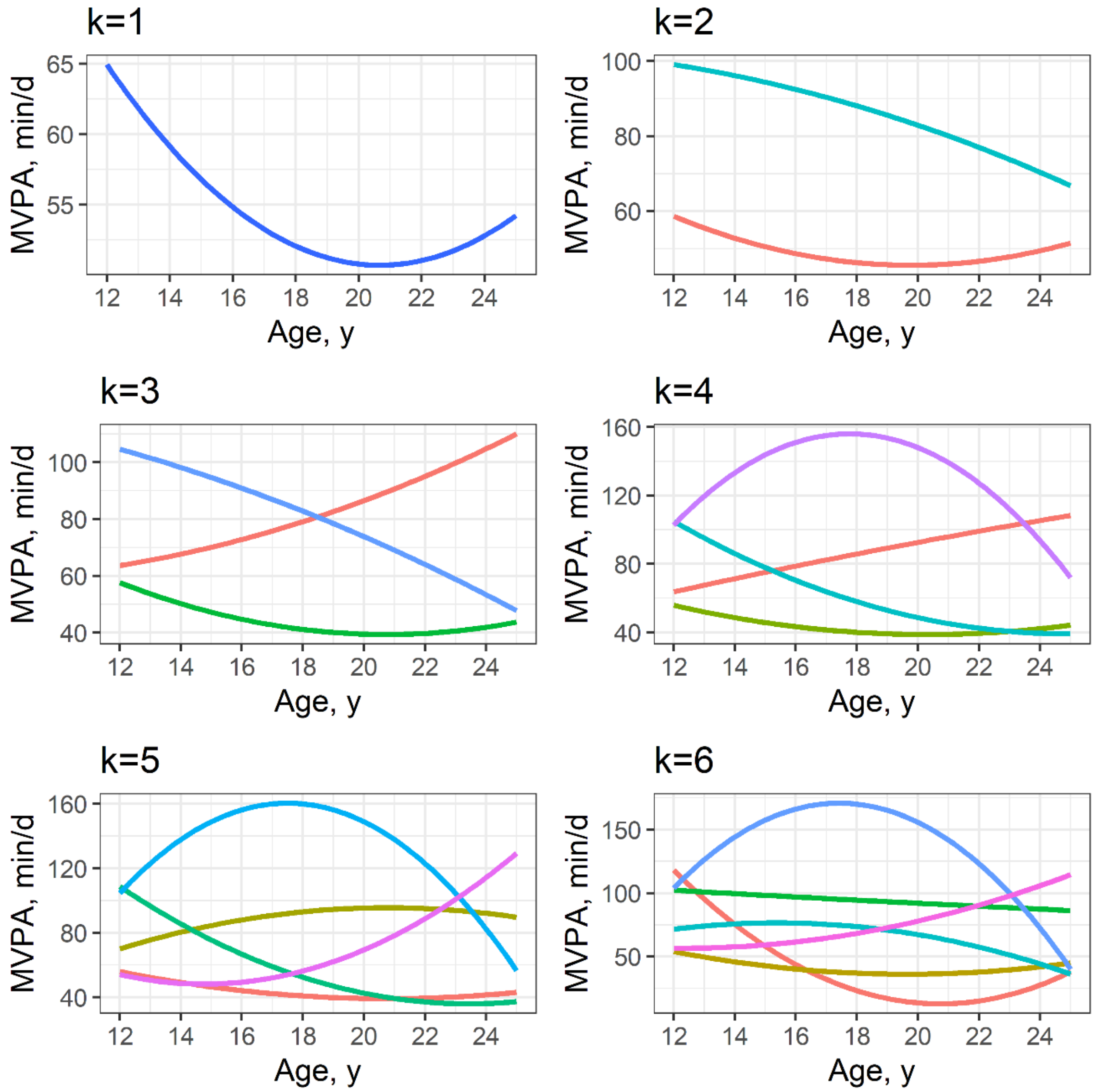

© 2020 Elhakeem A et al. *JAMA Network Open*.

eFigure 3. LPA Trajectories From Initial Latent Trajectory Models With Varying Number of Classes: Male Participants

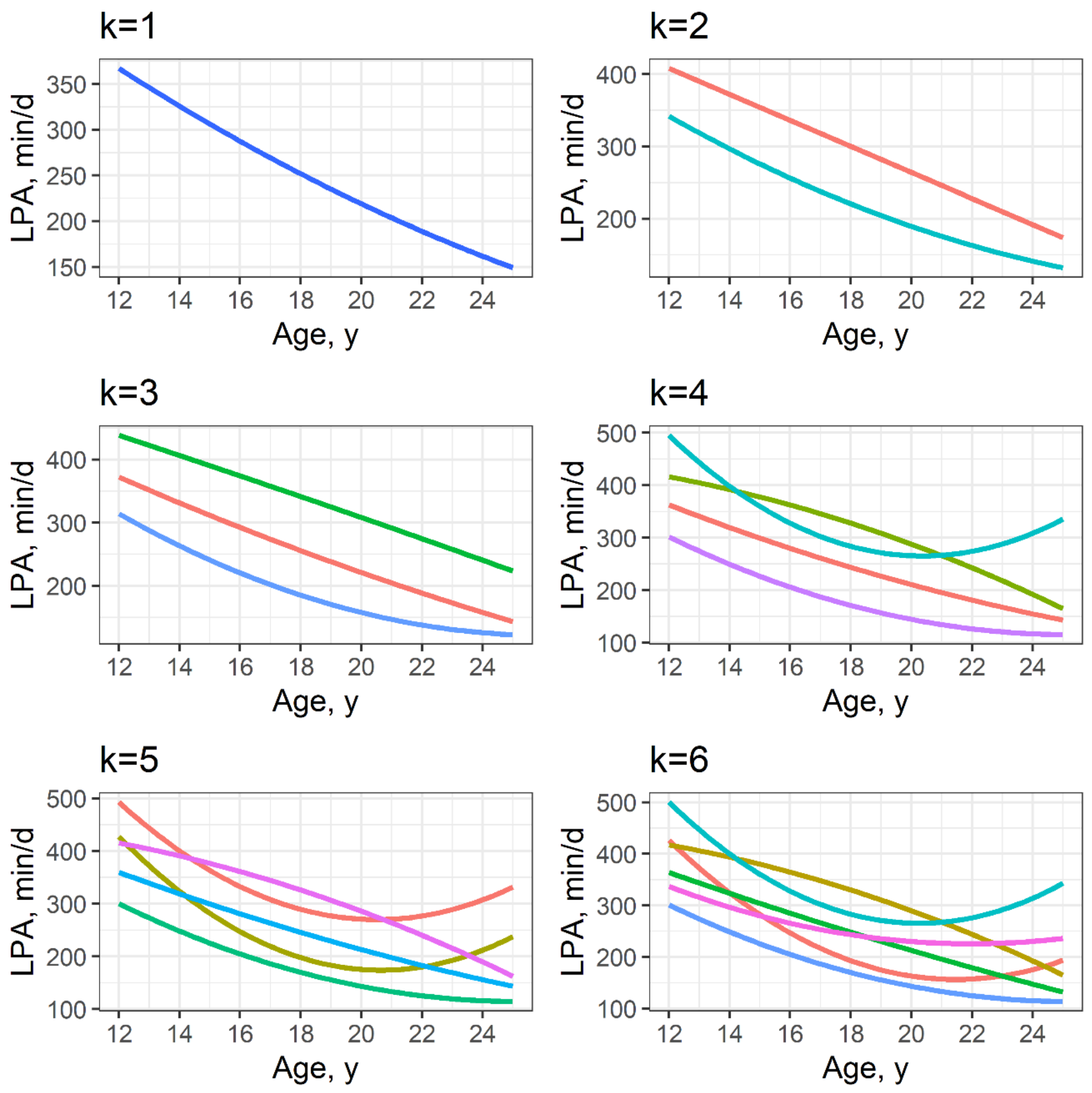

© 2020 Elhakeem A et al. *JAMA Network Open*.

eFigure 4. MVPA Trajectories From Initial Latent Trajectory Models With Varying Number of Classes: Female Participants

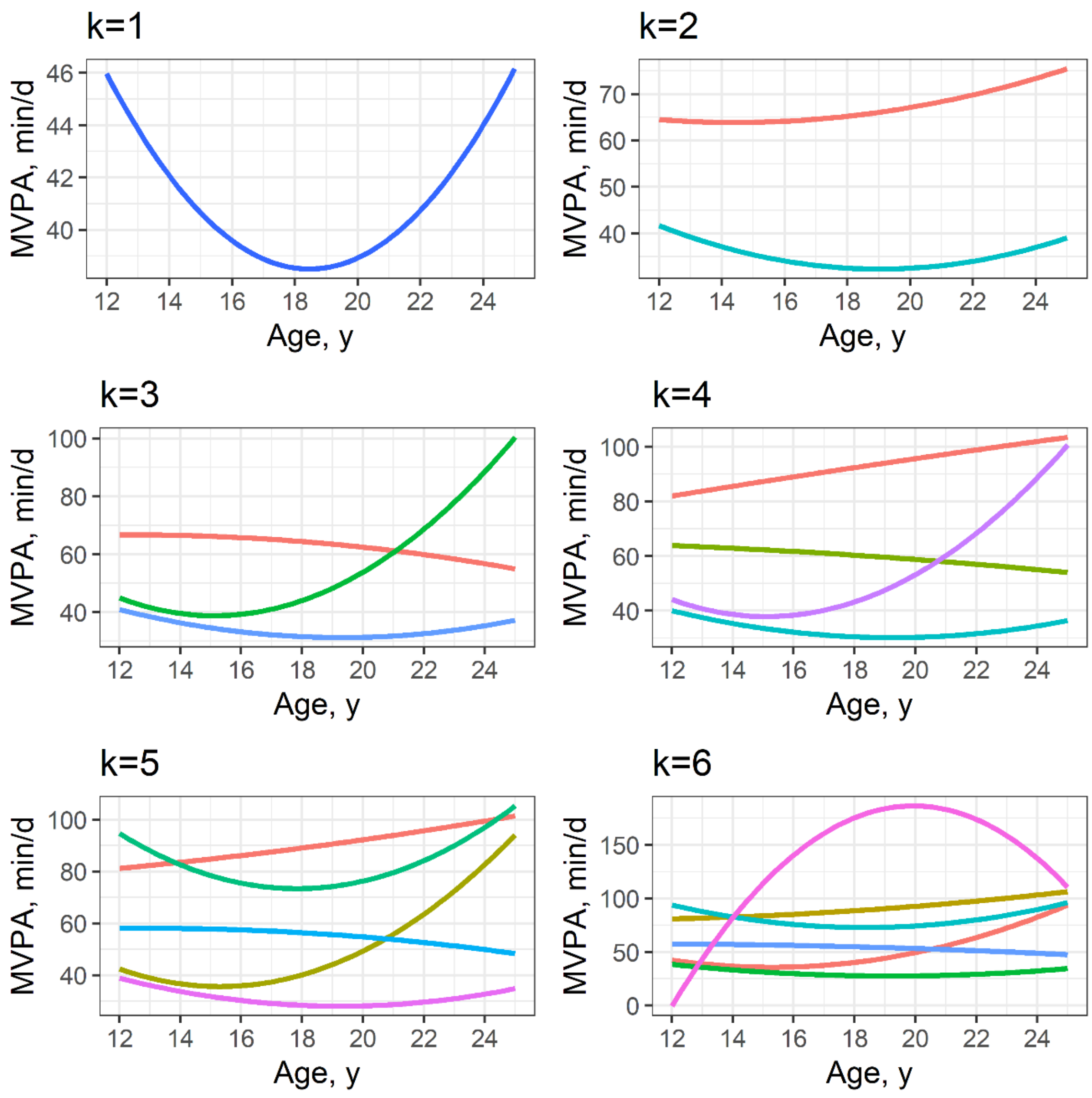

© 2020 Elhakeem A et al. *JAMA Network Open*.

eFigure 5. LPA Trajectories From Initial Latent Trajectory Models With Varying Number of Classes: Female Participants

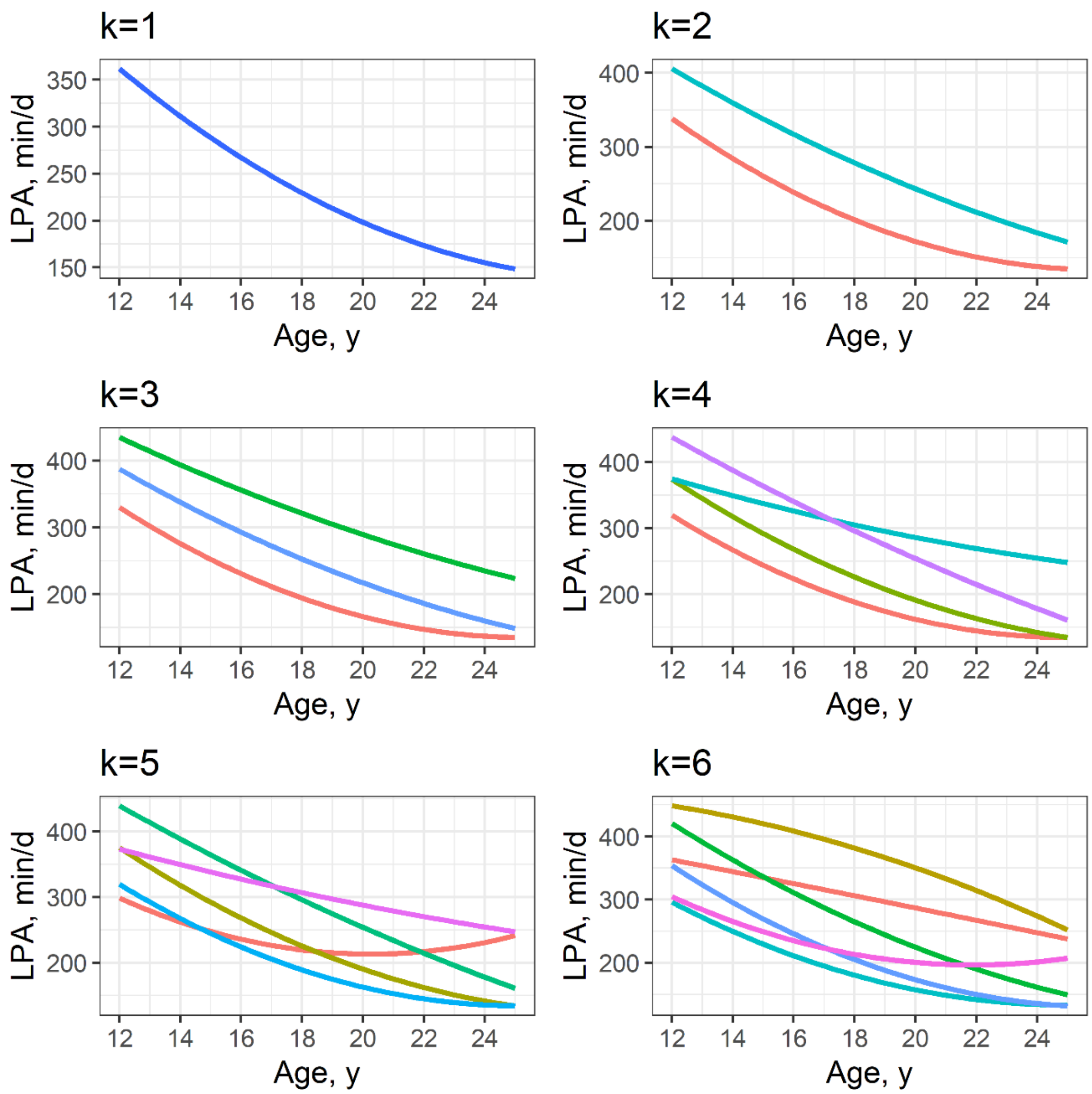

© 2020 Elhakeem A et al. *JAMA Network Open*.

eFigure 6. MVPA Trajectories From 3-Class MVPA Latent Trajectory Models With Varying Internal Model Structure: Male Participants

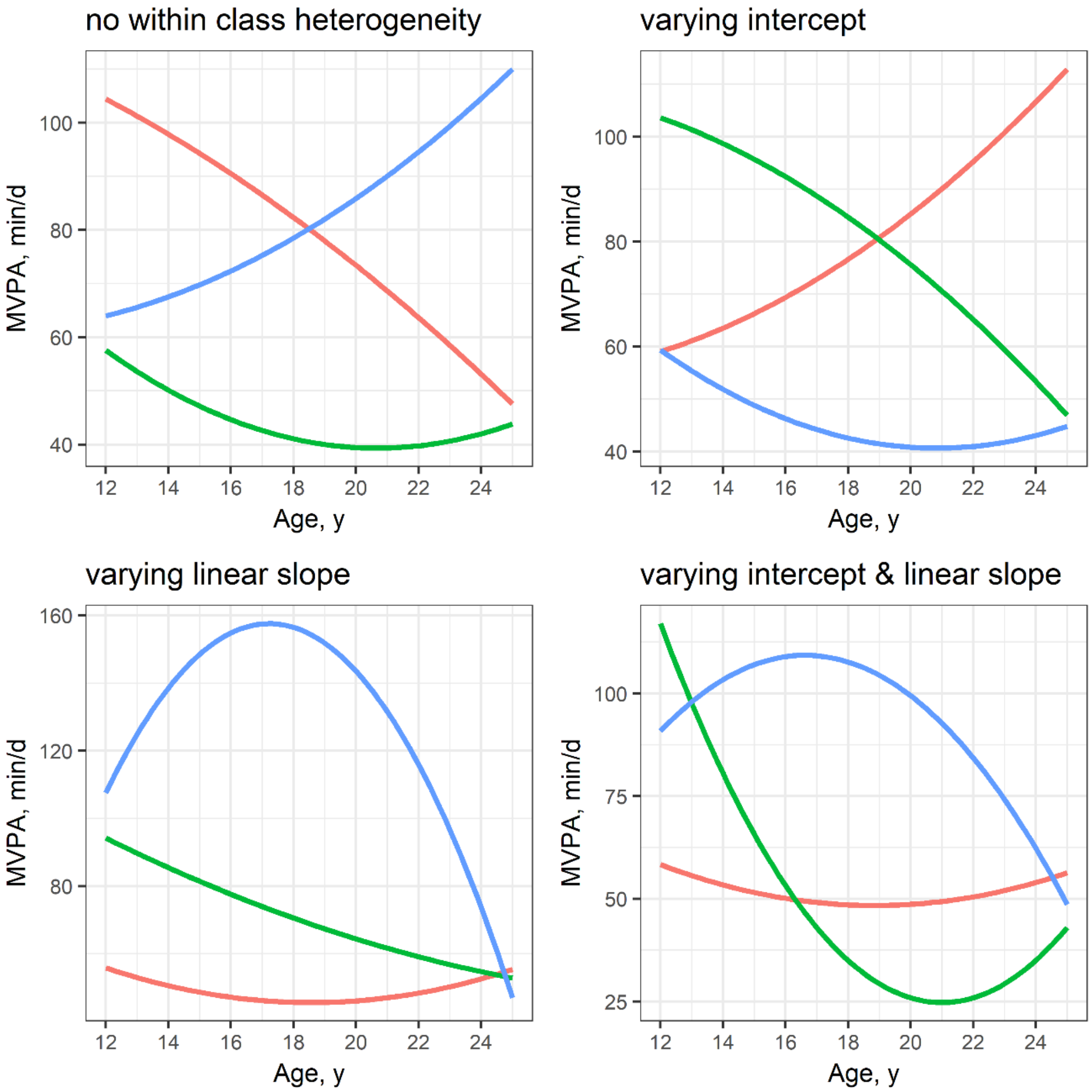

© 2020 Elhakeem A et al. *JAMA Network Open*.

eFigure 7. LPA Trajectories From 3-Class LPA Latent Trajectory Models With Varying Internal Model Structure: Male Participants

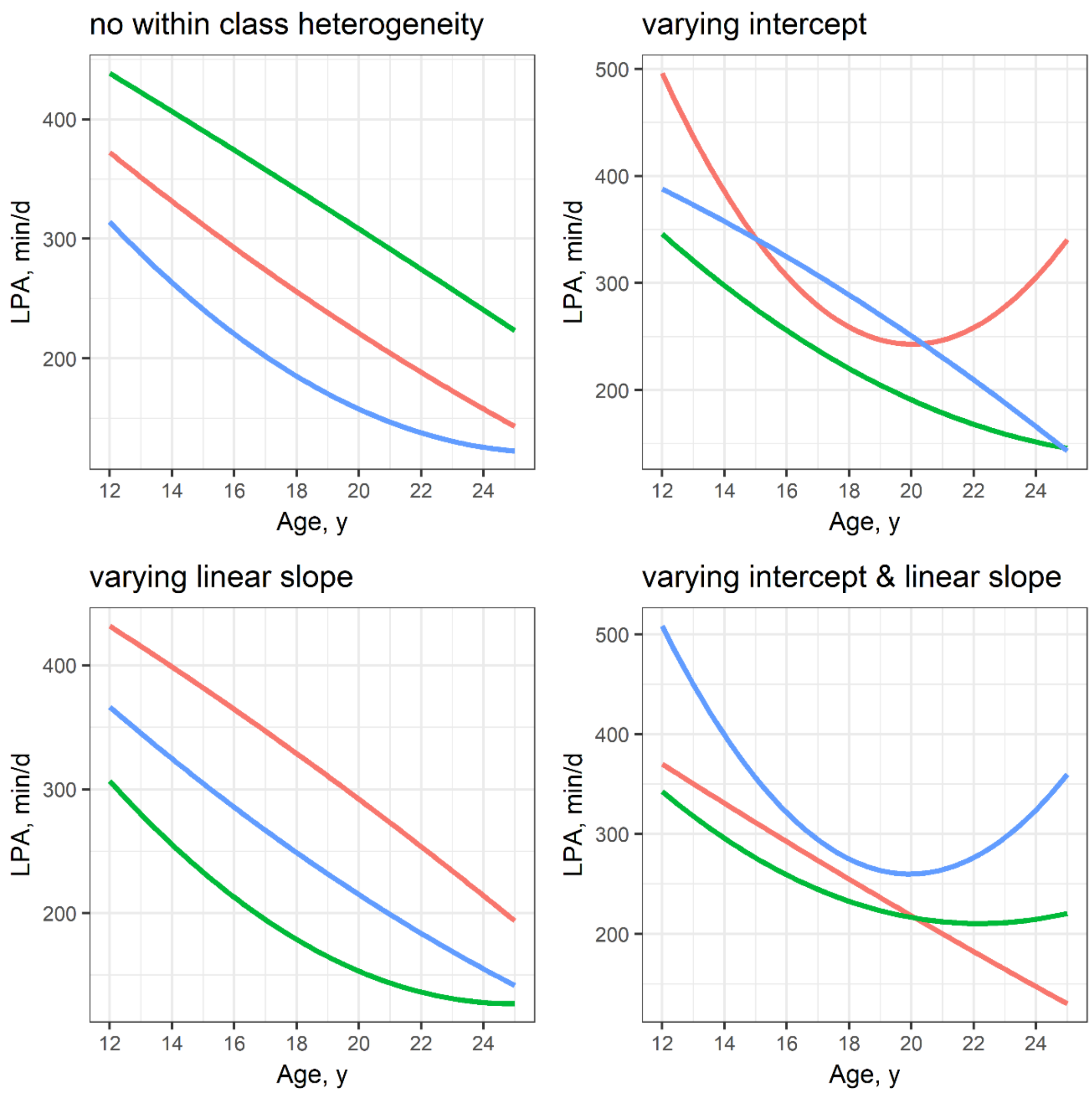

© 2020 Elhakeem A et al. *JAMA Network Open*.

eFigure 8. MVPA Trajectories From 3-Class MVPA Latent Trajectory Models With Varying Internal Model Structure: Male Participants

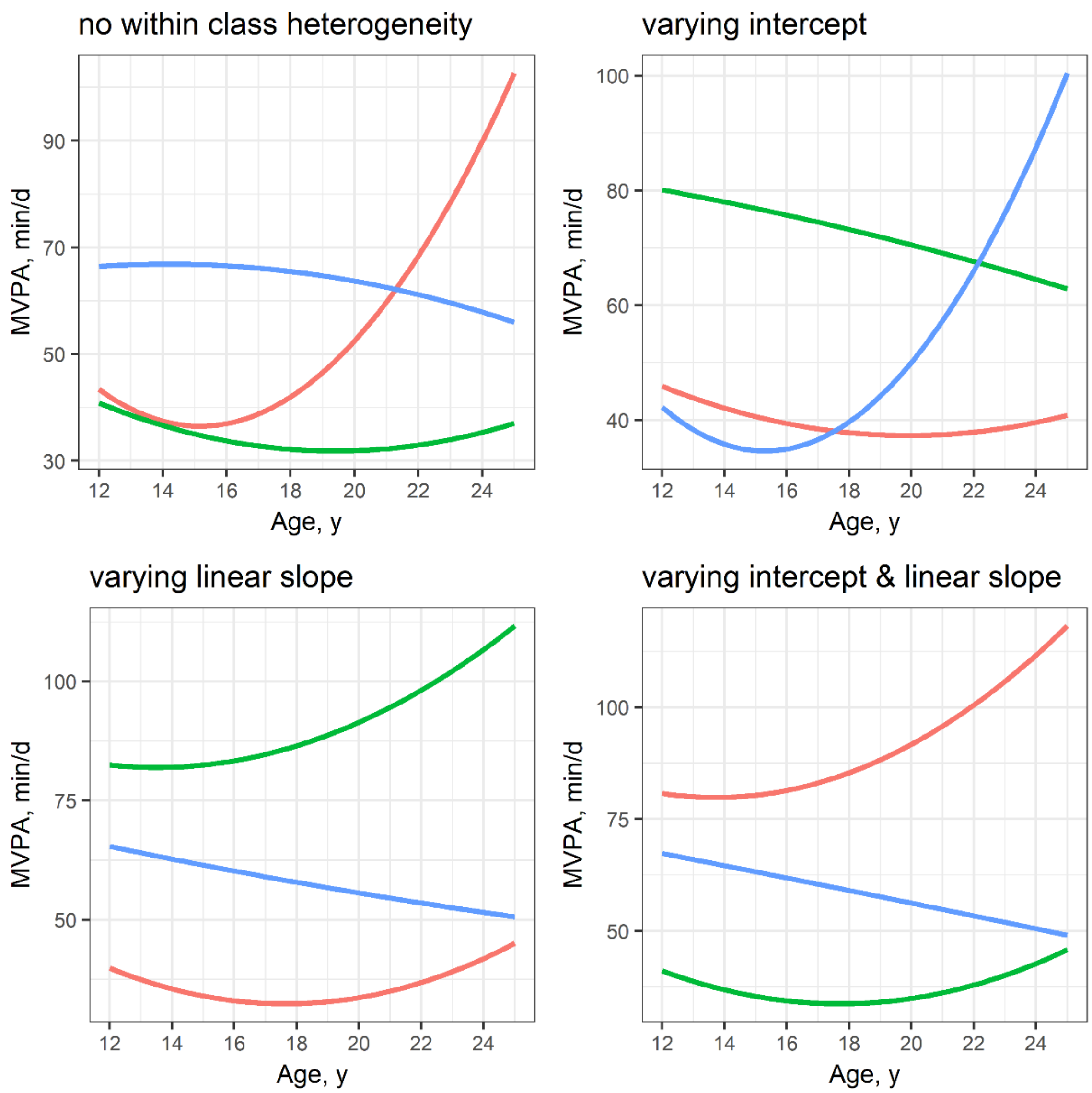

© 2020 Elhakeem A et al. *JAMA Network Open*.

eFigure 9. MVPA Trajectories From 3-Class MVPA Latent Trajectory Models With Varying Internal Model Structure: Male Participants

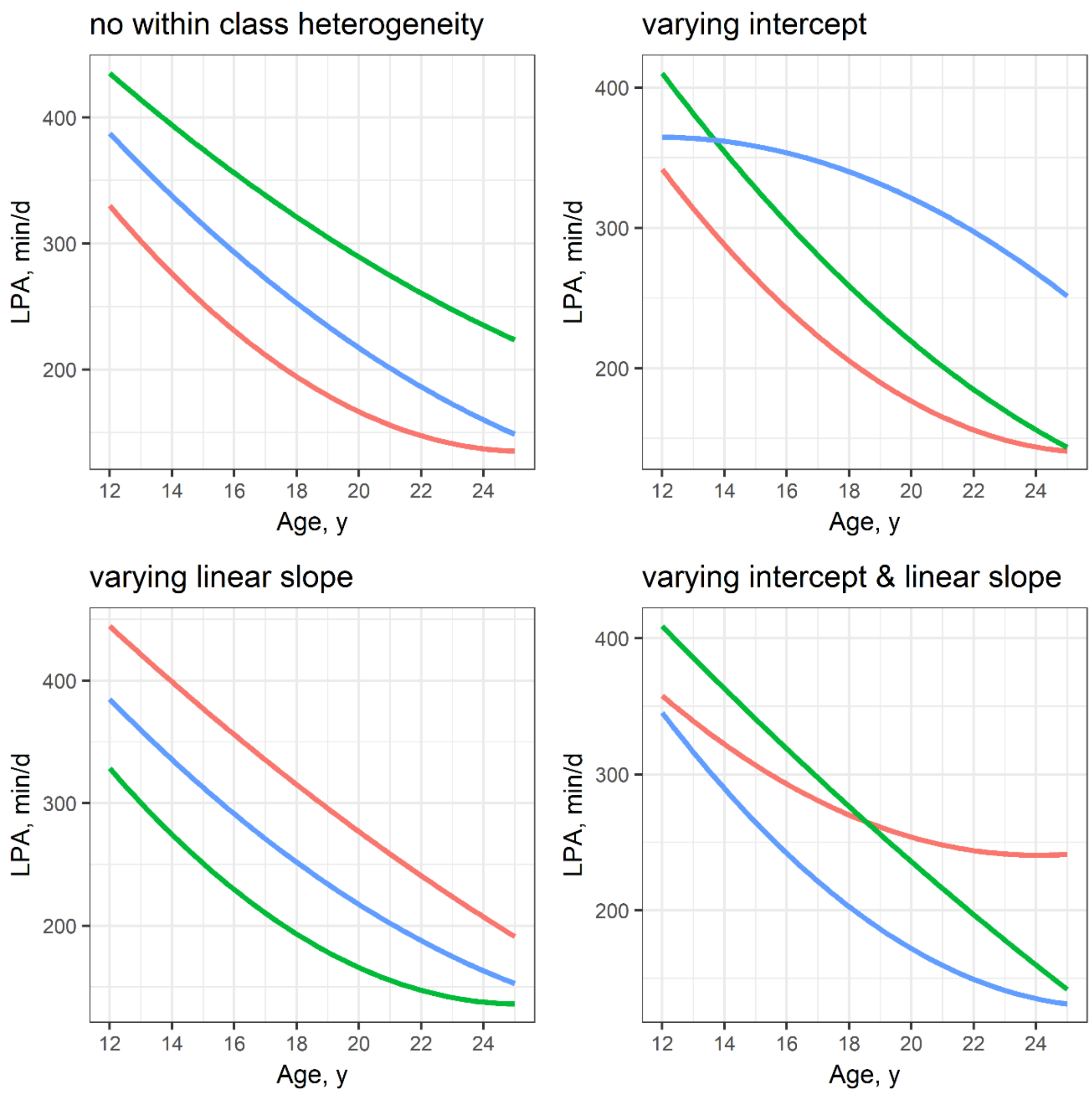

© 2020 Elhakeem A et al. *JAMA Network Open*.

eFigure 10. Observed MVPA and LPA Individual Trajectories by Most Likely Class From the Final 3-Class MVPA and LPA Trajectory Models

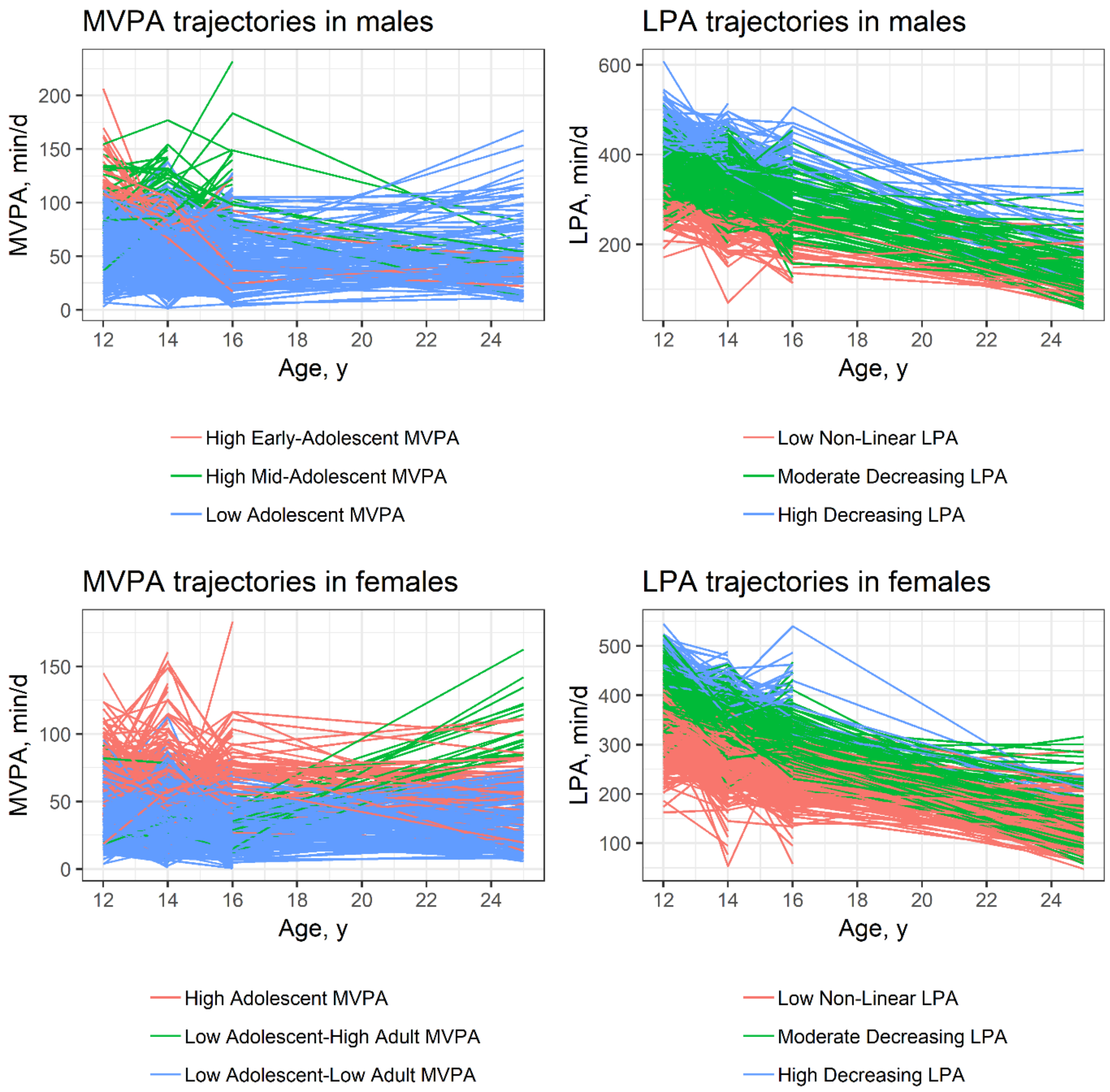

© 2020 Elhakeem A et al. *JAMA Network Open*.

eFigure 11. Physical Activity Trajectories From the Final 3-Class MVPA and LPA Latent Trajectory Models When Derived in the Maximum Sample Size (Not Restricted to Those With Complete Data on Confounders and Hip Outcomes)

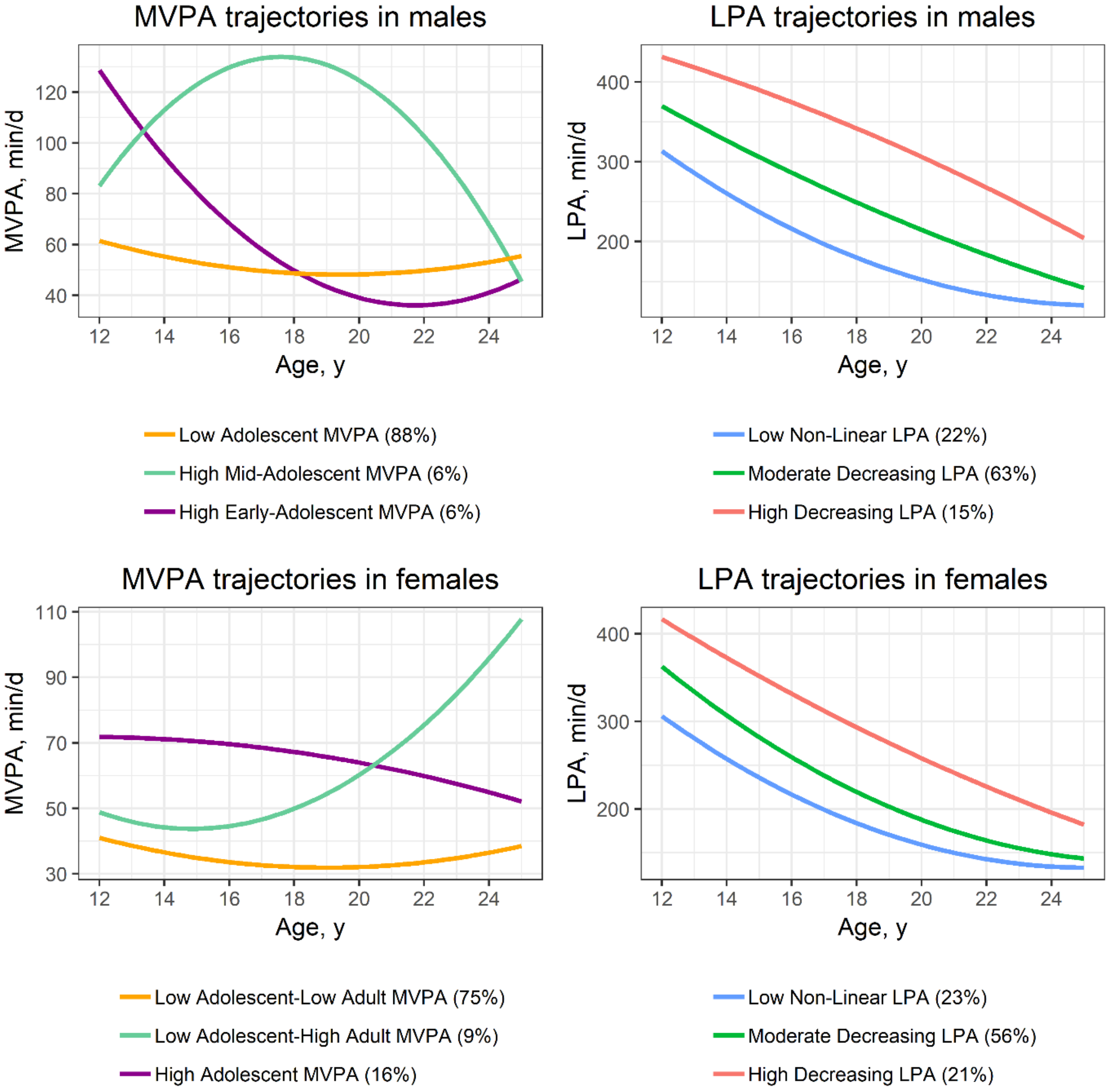

n=3234 males; n=3547 females

© 2020 Elhakeem A et al. *JAMA Network Open*.

eFigure 12. MVPA/LPA Missing Data Patterns and Proportions

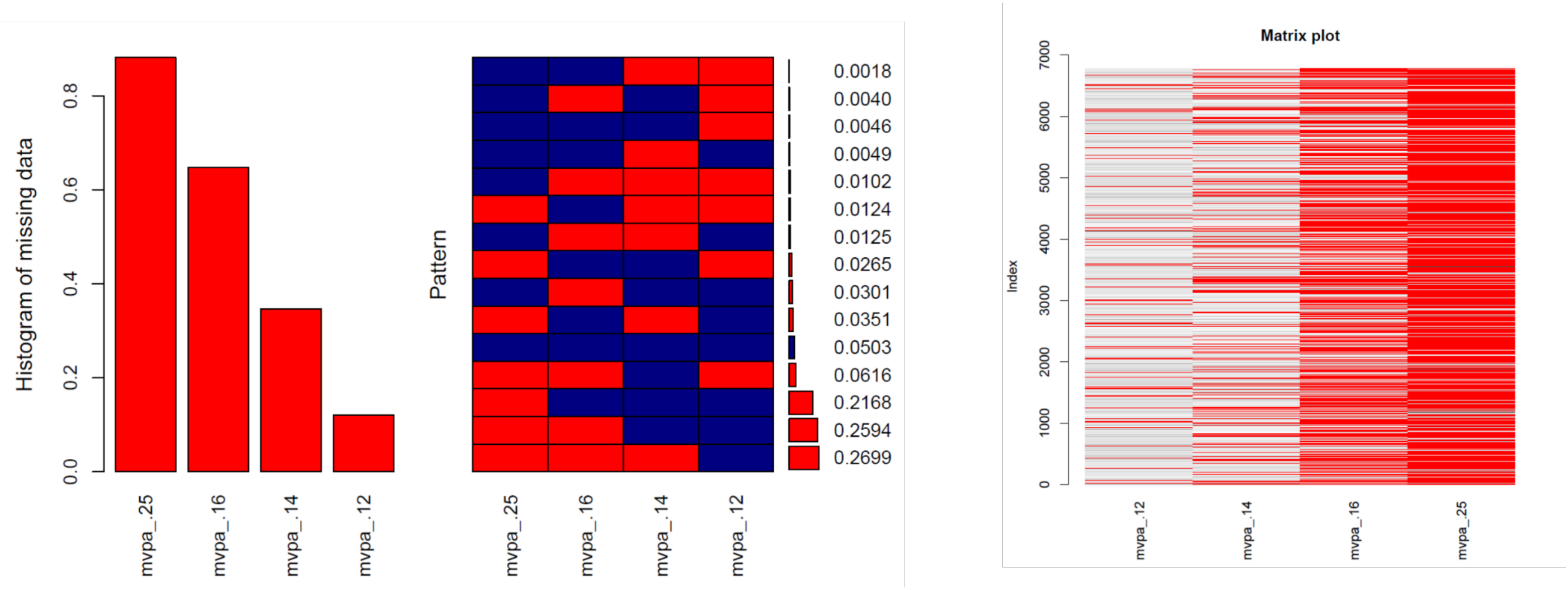

This plot gives the proportion of missingness in MVPA/LPA at each age (left panel), frequency of different combination of missing MVPA/LPA at different ages (middle panel), and a matrix plot showing frequency of missing MVPA/LPA at each age. Data shown for MVPA but the same applies to LPA.

© 2020 Elhakeem A et al. *JAMA Network Open*.

eFigure 13. Causal Diagram Depicting the Assumptions of a Negative-Outcome Control Study to Evaluate the Association of Physical Activity Throughout Adolescence on Peak Hip Strength in Young Adults

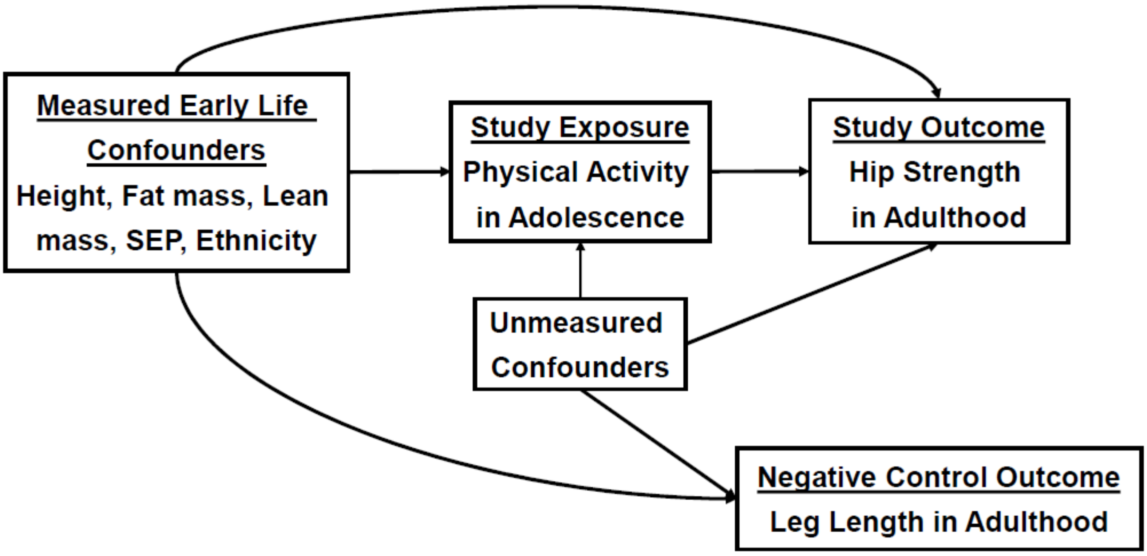

Adult leg length (the negative control outcome) should ideally have the same measured and unmeasured confounders as hip strength, but a causal link between adolescent physical activity and adult leg length is assumed implausible. SEP: socioeconomic position.

© 2020 Elhakeem A et al. *JAMA Network Open*.

eFigure 14. Scatter Plot of Observed MVPA and LPA by Age and Sex

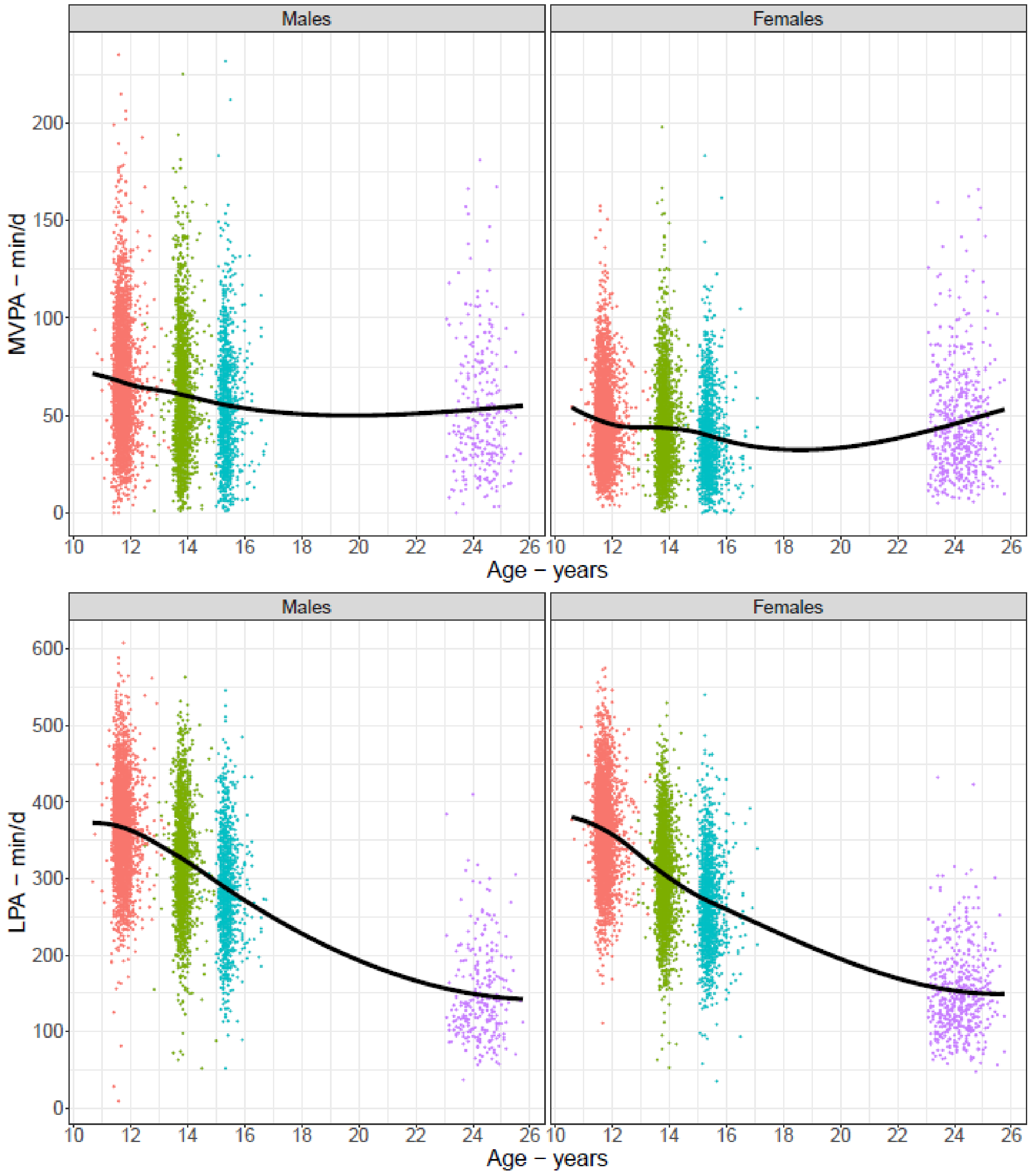

Colours represent observed values at each timepoint i.e. at ages 12, 14 16 and 25 years. Black lines represent Locally Weighed Scatterplot Smoothing (LOESS) curves.

© 2020 Elhakeem A et al. *JAMA Network Open*.

eFigure 15. Association of MVPA and LPA Trajectory and Physical Activity Gravitational Impacts With Adult Leg Length (the Negative-Outcome Control)

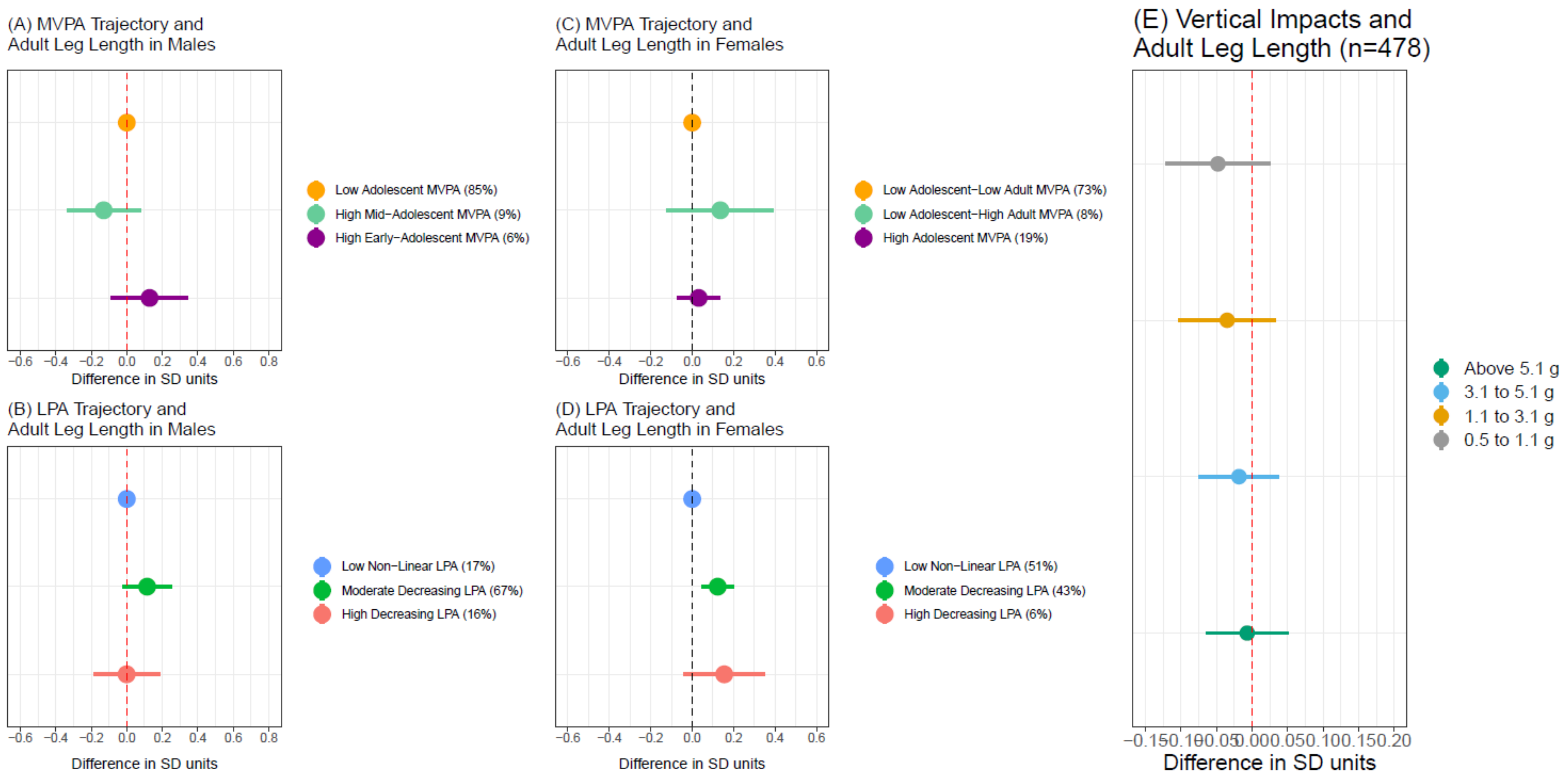

Estimates adjusted for ethnicity, maternal education, child height, fat and lean mass indices, and age at hip scan. Impacts results also adjusted for sex. For comparison, the results are shown on same axis scale as the adult hip strength results' figures in the main paper.

© 2020 Elhakeem A et al. *JAMA Network Open*.

eReferences

1. Lennon H, Kelly S, Sperrin M, Buchan I, Cross AJ, Leitzmann M, et al. Framework to construct and interpret latent class trajectory modelling. BMJ open. 2018;8(7):e020683.
2. Berlin KS, Parra GR, Williams NA. An Introduction to Latent Variable Mixture Modeling (Part 2): Longitudinal Latent Class Growth Analysis and Growth Mixture Models. Journal of pediatric psychology. 2013;39(2):188-203.
3. Jung T, Wickrama KAS. An Introduction to Latent Class Growth Analysis and Growth Mixture Modeling. Social and Personality Psychology Compass. 2008;2(1):302-17.
4. Ram N, Grimm KJ. Growth Mixture Modeling: A Method for Identifying Differences in Longitudinal Change Among Unobserved Groups. Int J Behav Dev. 2009;33(6):565-76.
5. Curran PJ, Hussong AM. The use of latent trajectory models in psychopathology research. Journal of abnormal psychology. 2003;112(4):526-44.
6. Kwong ASF, Lopez-Lopez JA, Hammerton G, Manley D, Timpson NJ, Leckie G, et al. Genetic and Environmental Risk Factors Associated With Trajectories of Depression Symptoms From Adolescence to Young Adulthood. JAMA Netw Open. 2019;2(6):e196587.
7. Kandola, A, Lewis G, Osborn DPJ, Stubbs B, Hayes FH. Depressive symptoms and objectively measured physical activity and sedentary behaviour throughout adolescence: a prospective cohort study. Lancet Psychiatry. 2020; doi:10.1016/S2215-0366(20)30034-1.
8. Herle M, Micali N, Abdulkadir M, Loos R, Bryant-Waugh R, Hübel C, et al. Identifying typical trajectories in longitudinal data: modelling strategies and interpretations. Eur J Epidemiol. 2020; doi:10.1007/s10654-020-00615-6
9. Lipsitch M, Tchetgen Tchetgen E, Cohen T. Negative controls: a tool for detecting confounding and bias in observational studies. Epidemiol. 2010;21(3):383-8.
10. Lawlor DA, Tilling K, Davey Smith G. Triangulation in aetiological epidemiology. Int J Epidemiol. 2016;45(6):1866-86.
11. Hamer M, Bauman A, Bell JA, Stamatakis E. Examining associations between physical activity and cardiovascular mortality using negative control outcomes. Int J Epidemiol. 2019. 48 (4), 1161-1166

© 2020 Elhakeem A et al. *JAMA Network Open*.

12. James P, Hart JE, Banay RF, Laden F. Exposure to greenness and mortality in a nationwide prospective cohort study of women. Environ Health Perspect. 2016;124(9):1344-52.
13. Gunnell DJ, Davey Smith G, Frankel SJ, Kemp M, Peters TJ. Socio-economic and dietary influences on leg length and trunk length in childhood: a reanalysis of the Carnegie (Boyd Orr) survey of diet and health in prewar Britain (1937-39). Paediatr Perinat Epidemiol. 1998;12 Suppl 1:96-113.
14. Li L, Dangour AD, Power C. Early life influences on adult leg and trunk length in the 1958 British birth cohort. Am J Hum Biol. 2007;19(6):836-43.
15. Wadsworth ME, Hardy RJ, Paul AA, Marshall SF, Cole TJ. Leg and trunk length at 43 years in relation to childhood health, diet and family circumstances; evidence from the 1946 national birth cohort. Int J Epidemiol. 2002;31(2):383-90.
16. Bogin B. Patterns of Human Growth. 2nd ed. Cambridge University Press; Cambridge, UK: 1999.
17. Bogin B, Varela-Silva MI. Leg length, body proportion, and health: a review with a note on beauty. Int J Environ Res Public Health. 2010;7(3):1047-1075. doi:10.3390/ijerph7031047

© 2020 Elhakeem A et al. *JAMA Network Open*.
